# Reproductive isolation arises during laboratory adaptation to a novel hot environment

**DOI:** 10.1186/s13059-024-03285-9

**Published:** 2024-05-28

**Authors:** Sheng-Kai Hsu, Wei-Yun Lai, Johannes Novak, Felix Lehner, Ana Marija Jakšić, Elisabetta Versace, Christian Schlötterer

**Affiliations:** 1 Institut für Populationsgenetik, Vetmeduni Vienna, Vienna, Austria; 2 Vienna Graduate School of Population Genetics, Vetmeduni Vienna, Vienna, Austria; 3 Institute of Animal Nutrition and Functional Plant Compounds, Vetmeduni Vienna, Vienna, Austria; 4 Present Address: École Polytechnique Fédérale de Lausanne, Lausanne, Switzerland; 5 Department of Biological and Experimental Psychology, Queen Mary University of London, London, UK

**Keywords:** *Drosophila simulans*, Experimental evolution, Mutation order speciation, Ecological speciation

## Abstract

**Background:**

Reproductive isolation can result from adaptive processes (e.g., ecological speciation and mutation-order speciation) or stochastic processes such as “system drift” model. Ecological speciation predicts barriers to gene flow between populations from different environments, but not among replicate populations from the same environment. In contrast, reproductive isolation among populations independently adapted to the same/similar environment can arise from both mutation-order speciation or system drift.

**Results:**

In experimentally evolved populations adapting to a hot environment for over 100 generations, we find evidence for pre- and postmating reproductive isolation. On one hand, an altered lipid metabolism and cuticular hydrocarbon composition pointed to possible premating barriers between the ancestral and replicate evolved populations. On the other hand, the pronounced gene expression differences in male reproductive genes may underlie the postmating isolation among replicate evolved populations adapting to the same environment with the same standing genetic variation.

**Conclusion:**

Our study confirms that replicated evolution experiments provide valuable insights into the mechanisms of speciation. The rapid emergence of the premating reproductive isolation during temperature adaptation showcases incipient ecological speciation. The potential evidence of postmating reproductive isolation among replicates gave rise to two hypotheses: (1) mutation-order speciation through a common selection on early fecundity leading to an inherent inter-locus sexual conflict; (2) system drift with genetic drift along the neutral ridges.

**Supplementary Information:**

The online version contains supplementary material available at 10.1186/s13059-024-03285-9.

## Introduction

Adaptation plays an important role in speciation [1–3] as already suggested by Darwin [4]. Two different mechanisms by which selection can lead to speciation have been proposed, ecological speciation and mutation-order speciation. During ecological speciation, reproductive isolation occurs as a consequence of adaptation to different ecological stressors. Importantly, reproductive isolation is not the direct target of selection, but rather occurs because the selected genes either have pleiotropic effects on reproduction or are linked to other loci affecting reproduction [5]. Mutation-order speciation describes the random occurrence of different favorable, but incompatible, mutations in distinct populations adapting to the same environmental stressor [6, 7]. Mutation-order speciation can result from intra-genomic conflict as well as antagonistic co-evolution between the two sexes [2]. Many examples of ecological speciation are documented [e.g., 8–13] and mutation-order speciation has received some empirical support [14].

On the other hand, it has been also proposed that speciation can result from stochastic processes (i.e., genetic drift) alone. Peak shift models are among the best-studied neutral speciation models. Despite some improvements, such as the “holey adaptive landscape” model [15], these models were criticized [16]. While there was a consensus that selection plays a much more important role in speciation than genetic drift [1], the recently developed model of system drift revives genetic drift as a potentially mechanism of speciation. Populations transverse along the neutral ridges by genetic drift and eventually develop incompatibilities, especially in small and isolated populations [17, 18].

These speciation models make different predictions for populations evolving independently in the same environments. Ecological speciation predicts that reproductive isolation develops between populations adapted to different environments, but not among different populations in the same environment [2]. With mutation-order speciation and system drift, independent populations which are adapted to the same environment become incompatible because they use different sets of incompatible mutations.

Experimental evolution provides an excellent empirical approach to study speciation processes [reviewed in: 19, 20], because environmental conditions can be well-controlled and replicate populations are exposed to the same environmental stress. Nevertheless, only ecological speciation has received strong empirical support by laboratory experiments [21–24], and in many cases, the selected traits were not identified.

Here, we take advantage of a highly replicated experimental evolution study, where 10 replicate populations derived from a single polymorphic *D. simulans* population have adapted independently to a novel hot temperature regime in about 100 generations [25, 26]. Although ambient temperature is one of the major ecological factors driving adaptation in natural populations, it is not clear from previous work to what extent temperature has triggered speciation processes [27]. We find support for (incomplete) premating isolation between ancestral and hot-evolved populations, but no difference was found among the replicates, which evolved independently in the same novel hot environment. Interestingly, we also observed some evidence for postmating reproductive isolation between the evolved replicates. We propose that premating reproductive isolation is based on a modified lipid metabolism, which also causes shifts in CHC composition, a well-understood mechanism of reproductive isolation in fruit flies [28–33]. We suggest that the high variance in evolutionary expression change of male reproduction-related genes may underlie the observed postmating reproductive isolation.

## Results

**Fig. 1:**
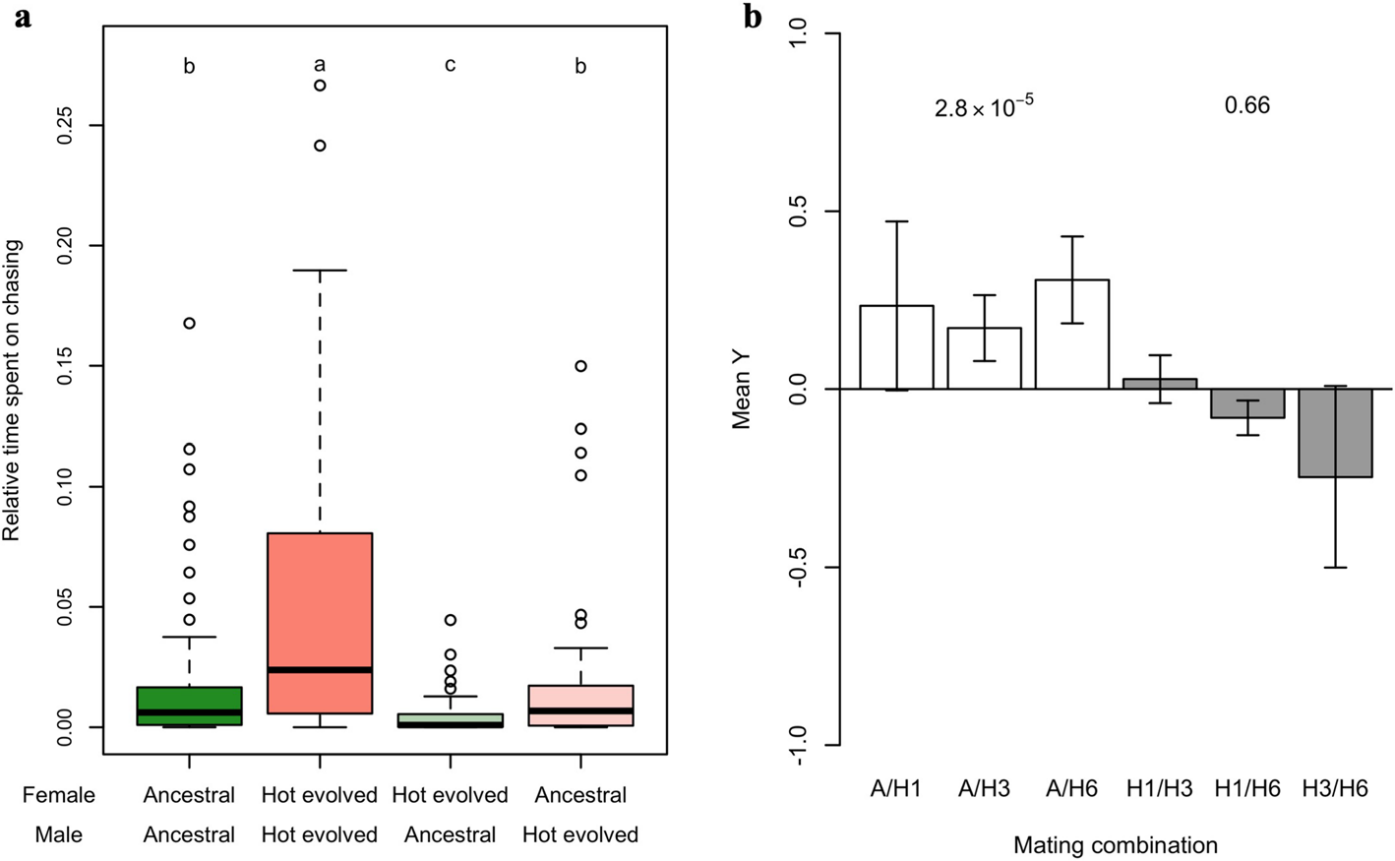
Evolution of mating preference and premating reproductive isolation. **a** Male reproductive activity in the presence of the females from the same evolved population or the ancestral one. The reproductive activity was measured as the time spent on chasing. Males spent significantly more time chasing females from the same populations (Kruskal–Wallis test, *p* < 0.001; post-hoc groups were indicated as the small-case letter above the boxes). **b** Yule’s index for assortative mating. A denotes ancestral population; H1, H3, and H6 denote the three randomly selected hot-evolved replicates included in the multiple choice mating assay. There is significant positive assortative mating between flies from the same populations between ancestral and evolved populations (white bars) while there’s no significant effect among the replicate evolved populations (*p*-values for Fisher’s exact test)

After more than 100 generations of adaptation to a novel high temperature regime, we measured reproductive isolation. We observed significant mate discrimination between ancestral and hot-evolved populations but not for replicate populations evolved independently to the same selection regime. The courting behavior of evolved males was significantly reduced when exposed to ancestral females and the same reduction was seen for males from the ancestral population in combination with hot-evolved females (Fig. 1a, Kruskal–Wallis test, *p* < 0.05). Reproductive isolation was further shown in an assortative mating experiment where flies from two populations were mixed and mating partners could be chosen freely. Combining ancestral and evolved populations resulted in strong positive assortative mating, while no assortative mating could be detected among independently evolved populations (Fig. 1b). These patterns of premating reproductive isolation are fully consistent with the expectations for ecological speciation: significant differences between flies adapted to different environments, but no reproductive isolation between flies from different populations evolved in the same environment.

**Fig. 2:**
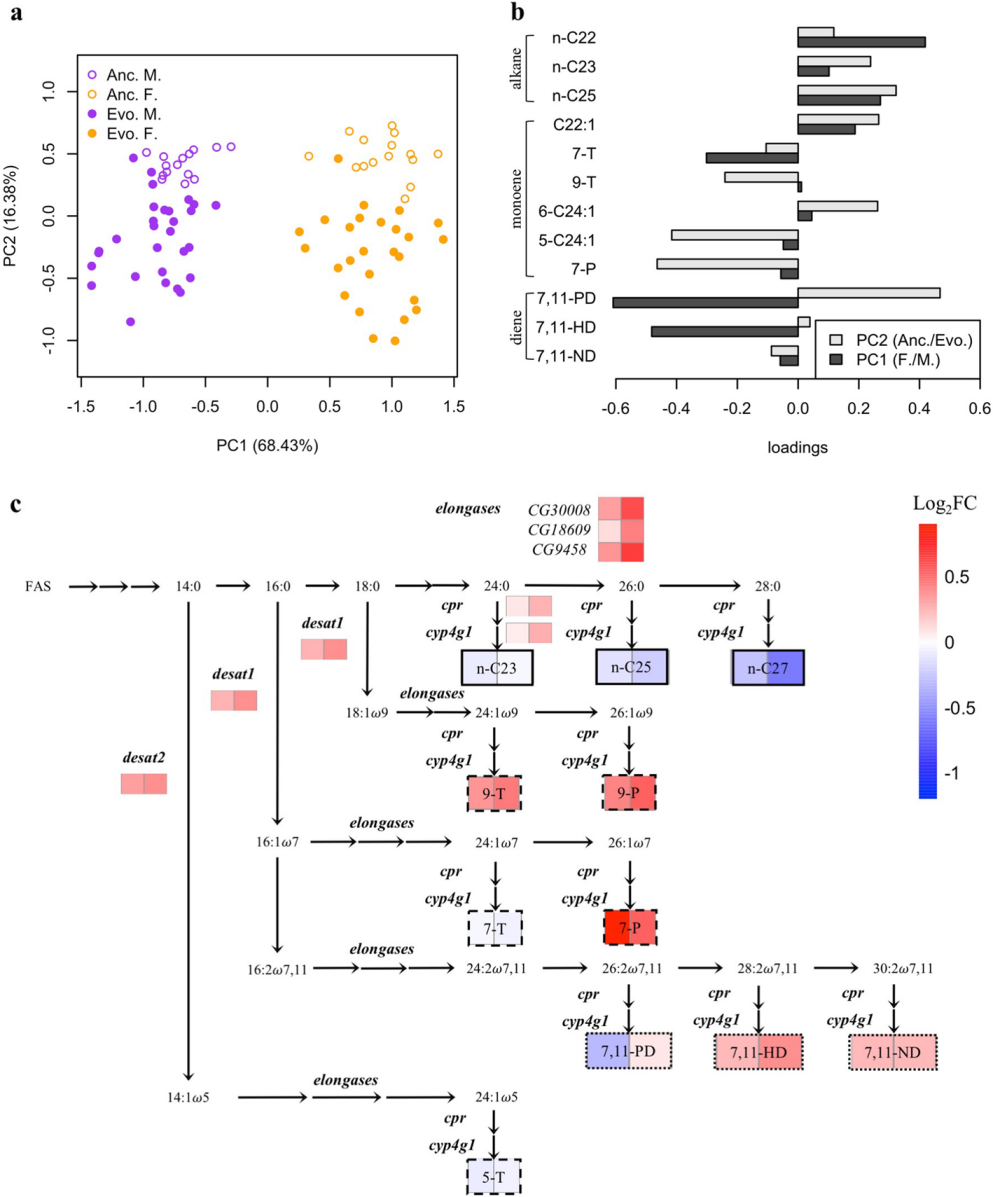
Evolution of cuticle hydrocarbons (CHCs) metabolism. **a** Principal component analysis on the center-log transformed relative abundance of 12 CHCs detected in all samples. (orange: females; purple: males). Solid dots are evolved samples and empty ones are ancestral samples. **b** Loading of each compound on the first and second principal components (PCs). Dark gray bars indicate the loadings for PC1. Positive loading suggests higher abundance in females. Light gray bars indicate the loadings for PC2 and positive values reflect higher abundance in the ancestral samples. **c** Evolutionary changes in gene expression of the biosynthetic pathway for *Drosophila* cuticular hydrocarbons (CHCs). The heat maps underneath each compound (boxes with regular fonts) or genes (boxes with italic fonts) denote the changes in abundance during adaptation (log2-scale). The left cell denotes the changes in females and right cell for males. Red indicates increase and blue indicates decrease in abundance

Previously we showed that the lipid metabolism is significantly altered in the hot-evolved populations studied here [25]. Since the synthesis of cuticular hydrocarbons (CHCs), which serve a central role in mate recognition [28–30], is controlled by genes involved in lipid metabolism [34], we reasoned that the CHC composition may have been altered as a byproduct of the change in lipid metabolism. We tested the divergence in CHC composition between ancestral and evolved populations by measuring all hydrophobic compounds on the outer skeleton of virgin flies of each sex with gas chromatography/MASS spectrometry (GC/MS). We detected 18 major CHC compounds across both sexes, which differ in length of carbon chain or numbers of double bonds [28, 34] (Table 1 and Additional file 1: Fig. S1). Principal component analysis (PCA) indicated that the relative abundance of the CHCs differed significantly between sexes as well as between evolved and ancestral populations (Fig. 2a). The first PC, which explains 68.4% of total variance, reflects the differences between the two sexes — males synthesize more unsaturated CHCs than females (Fig. 2b). The variation caused by adaptation to a hot laboratory environment is reflected by PC2 which accounts for 16.38% of the total variance (Fig. 2a). No significant interactions between sexual dimorphism and evolution were observed for these traits (Table 1), indicating parallel evolution of CHC composition in both sexes. Evolved flies synthesize more unsaturated CHCs with longer carbon chains (Fig. 2b and c). Interestingly, similar modifications in CHC compositions have been documented for multiple *Drosophila* species in latitudinal clines [35], implying that the same causal link with temperature adaptation is also present in natural populations.

**Table 1:** Abundance of cuticular hydrocarbons (CHCs) in relation to sex and evolution

Peak No	Retention index	Name	% in anc. female	% in anc. male	% in evo. female	% in evo. male	ANOVA (*p*-value)		
							**Sex**	**Evolution**	**Sex:evolution**
1	2187	x-Docosene (x-C22:1)	0.96 ± 0.02	0.69 ± 0.02	0.88 ± 0.02	0.66 ± 0.01	1.18E − 18	1.88E − 09	9.04E − 01
2	2195	cis-Vaccenyl Acetate (cVA)	-	-	-	-	-	-	-
3	2200	n-Docosane (n-C22)	1.35 ± 0.04	0.58 ± 0.01	1.26 ± 0.03	0.67 ± 0.03	1.37E − 31	1.38E − 02	3.40E − 01
4	2279	9-Tricosene (9-T)	1.67 ± 0.07	2.77 ± 0.09	2.16 ± 0.11	3.78 ± 0.09	1.99E − 27	1.23E − 04	6.95E − 01
5	2285	7-Tricosene (7-T)	75.48 ± 0.42	75.29 ± 0.44	73.64 ± 0.44	71.70 ± 0.52	2.64E − 01	1.01E − 06	6.02E − 01
6	2294	5-Tricosene (5-T)	0.68 ± 0.03	0.83 ± 0.04	0.64 ± 0.04	0.79 ± 0.02	1.04E − 03	1.47E − 03	5.59E − 01
7	2300	n-Tricosane (n-C23)	9.64 ± 0.20	8.39 ± 0.14	9.08 ± 0.15	8.14 ± 0.17	1.39E − 05	1.65E − 07	9.04E − 01
8	2383	6-Tetracosene (6-C24:1)	0.64 ± 0.01	0.58 ± 0.02	0.87 ± 0.03	0.86 ± 0.03	1.43E − 01	1.97E − 12	5.49E − 01
9	2391	5-Tetracosene (5-C24:1)	0.25 ± 0.01	0.27 ± 0.01	0.40 ± 0.02	0.42 ± 0.02	1.46E − 01	1.33E − 11	5.59E − 01
10	2400	n-Tetracosane (n-C24)	0.34 ± 0.02	0.13 ± 0.02	0.39 ± 0.02	0.15 ± 0.02	1.52E − 21	7.32E − 01	6.27E − 01
11	2465	7,11-Pentacosadiene (7,11-PD)	0.35 ± 0.03	0.82 ± 0.03	0.28 ± 0.02	0.86 ± 0.05	1.12E − 29	1.53E − 04	3.40E − 01
12	2478	9-Pentacosene (9-P)	0.95 ± 0.05	0.75 ± 0.04	1.28 ± 0.12	1.08 ± 0.05	1.79E − 06	9.40E − 06	6.08E − 01
13	2486	7-Pentacosene (7-P)	1.59 ± 0.06	1.98 ± 0.05	2.93 ± 0.18	2.90 ± 0.12	4.26E − 02	8.37E − 12	3.71E − 02
14	2500	n-Pentacosane (n-C25)	1.44 ± 0.05	1.00 ± 0.04	1.31 ± 0.03	0.85 ± 0.03	2.45E − 14	8.43E − 07	5.59E − 01
15	2665	7,11-Heptacosadiene (7,11-HD)	1.85 ± 0.07	4.04 ± 0.10	2.18 ± 0.08	5.28 ± 0.20	2.24E − 45	1.38E − 02	5.59E − 01
16	2700	n-Heptacosane (n-C27)	1.56 ± 0.08	0.68 ± 0.05	1.28 ± 0.09	0.45 ± 0.05	1.22E − 26	3.25E − 05	7.45E − 01
17	2863	7,11-Nonacosadiene (7,11-ND)	1.11 ± 0.06	1.20 ± 0.05	1.31 ± 0.06	1.41 ± 0.08	8.30E − 02	7.21E − 01	6.08E − 01
18	3000	n-Triacotane (n-C30)	-	-	-	-

To elucidate the basis of the CHC composition changes, we revisited the published gene expression data of the ancestral and replicates of the evolved populations from the same evolution experiment [25]. Among the 469 genes with a significant increase in expression in both sexes across 10 replicate evolved populations, we found a prominent enrichment for genes involved in “very long-chain fatty acid biosynthetic process.” Twenty-seven out of the 36 (33 are detected in our data) previously reported CHC synthesis-related genes in *Drosophila melanogaster* [34] showed significant expression changes in at least one sex during adaptation in the replicated *D. simulans* populations (Fisher’s exact test, odds ratio = 3.33, *p*-value < 0.01; Supplementary Figure 2). 16 of these genes changed their expression in the same direction in both sexes. Genes encoding desaturases (*desat 1* and *desat 2*) and elongases (*CG9458*, *CG18609*, and *CG30008*) were consistently up-regulated in all replicate evolved populations (Fig. 1c and Additional file 2: Fig. S2). This consistent transcriptional modification of long-chain fatty acid/CHC biosynthesis during the evolution can explain the significant changes in CHC composition in the evolved flies.

**Fig. 3:**
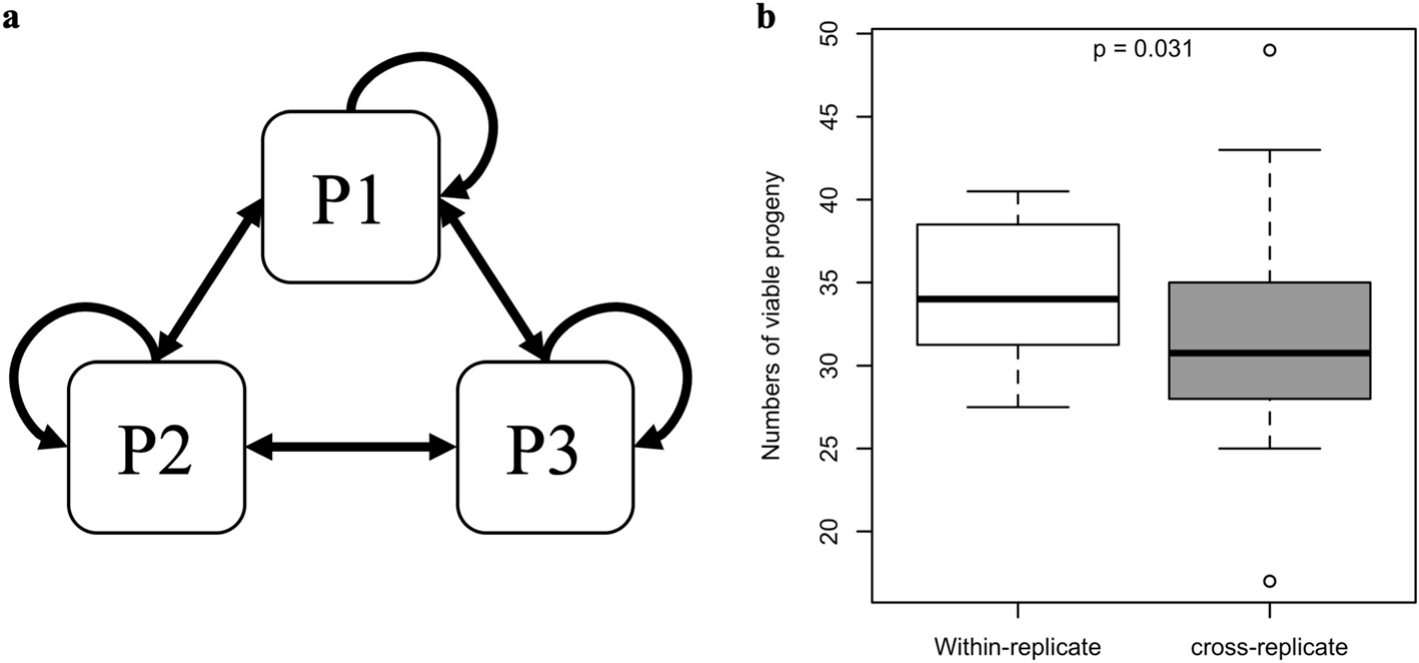
Evolution of postmating incompatibility among independently evolved populations. **a** Diallel cross design among three replicate evolved populations. Flies from each population were crossed with flies from the same population and the other populations reciprocally. **b** Fitness difference between within-replicate crosses and cross-replicate ones. The fitness was measured as the number of viable adults in each cross. Fitness was significantly higher in within-replicate crosses (*p* = 0.031, Wilcoxon’s test), indicating postmating incompatibility between independently evolved replicates

Reproductive isolation can occur before and after mating. We also tested postmating reproductive isolation among evolved replicates. Crosses between replicates of the evolved populations produced on average 8.3% fewer viable offspring than crosses within the same replicates of the evolved populations (Fig. 3b, Wilcoxon’s test, *p* = 0.031). Moreover, crosses between replicate populations never generated more offspring than mid-parent expectation (i.e., the mean number of offspring in the pure parental lines, Additional file 3: Fig. S3). Considering the absence of premating isolation (Fig. 1b), the reduced number of hybrid offspring indicates that another postmating process affecting fertilization and/or viability occurs between the ancestral and evolved populations and leads to reproductive isolation.

**Fig. 4:**
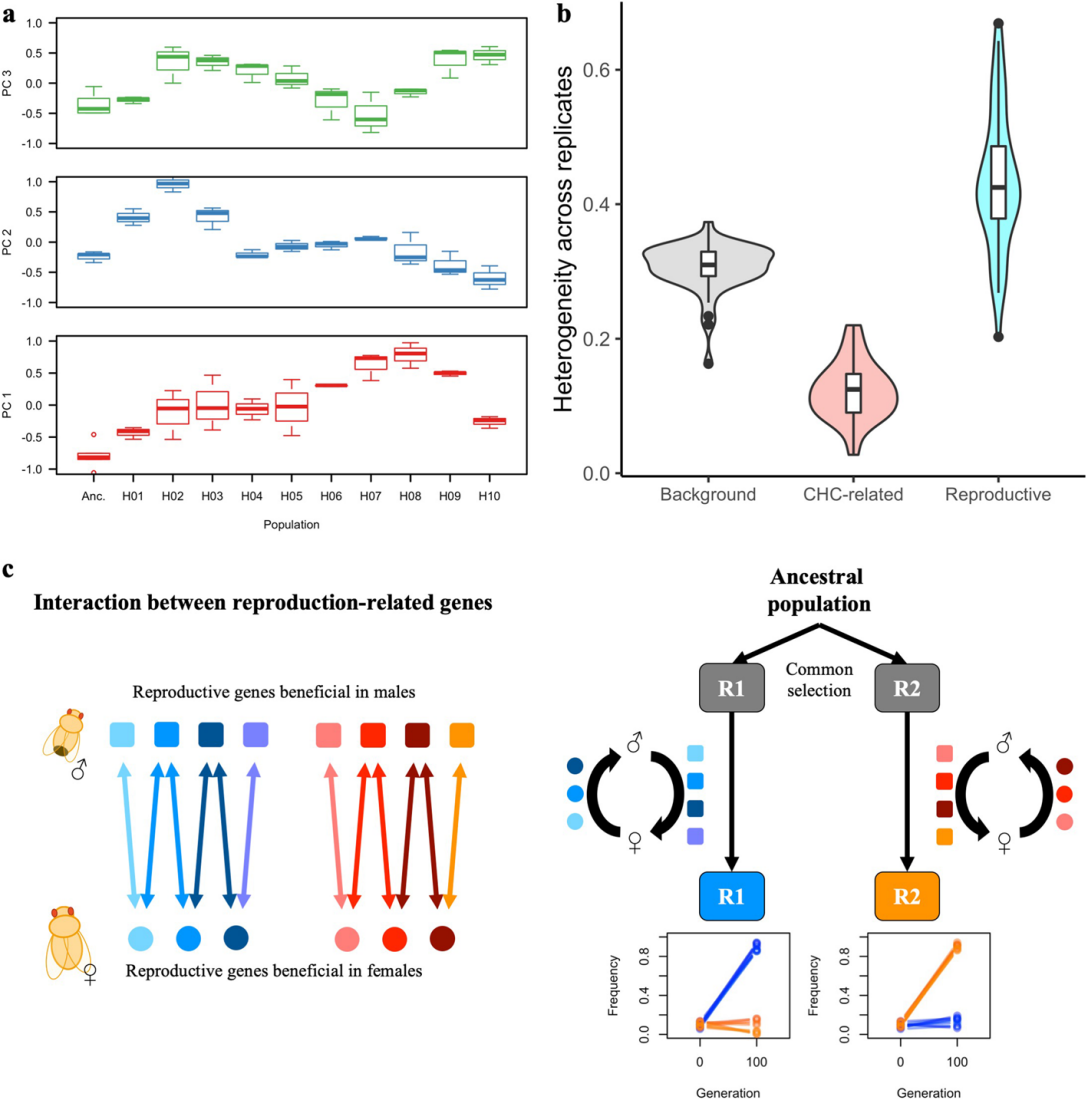
Heterogeneous adaptive changes in reproduction-associated genes** a** Replicate-specific evolution caused the expression divergence of reproduction-associated genes. The expression variance among 255 reproduction-associated genes (see the “Material and methods” section) is summarized by the leading principal components (PCs). The first three PCs suggest that different replicates evolved different sets of genes during adaptation. **b** The heterogeneity in expression evolution differs among different sets of genes: background (all expressed genes), CHC-related (33 genes reported in [34]), reproduction-associated (255 genes from GO term (GO:0032504)). The heterogeneity is measured as $\sum_{i,j}(1-\text{cor}\left({x}_{i},{x}_{j}\right))/N$, where *x* is a vector consisting of the expression of each gene in each evolution replicate; $i,j\in \left[\text{1,10}\right], i\ne j$ and *N* is the total number of pairwise combination of 10 replicates. Reproduction-associated genes evolved with significantly higher heterogeneity than background genes or CHC-associated genes (Kruskal–Wallis test, *p* < 0.001). **c** An illustration for how intersexual co-evolution could lead to heterogeneous and incompatible evolution in isolated populations. Genes involved in male reproduction also serve important functions in females via either synergistic or antagonistic interaction with female reproduction-associated genes. Multiple epistatic modules with different genes involved (red and blue colors) may co-exist in the ancestral population. A cascade of intersexual co-evolution for a certain group of genes is expected if selection pressure is imposed on either sex. As such co-evolutionary cascades (modules) are not unique, but redundant, replicate populations may evolve for high divergence and the alternative co-evolutionary paths may not be fully compatible with each other, resulting in fitness costs for between-replicate crosses

We used RNA-Seq to explore the functional basis of this putative postmating reproductive isolation. Reasoning that putative causal genes may diverge in gene expression, we searched for genes differentially expressed among replicate evolved populations. In total, we identified 3062 genes which differed in at least one evolved population from the others (Additional file 4 Fig. S4, Additional file 5: Table S1). Functional enrichment analysis revealed that a substantial portion of these divergently expressed genes is highly expressed in the testis and is involved in reproduction-associated processes (e.g., multicellular organism reproduction (GO:0032504); Additional file 6: Table S2). Focusing on 255 reproduction-associated genes that diverged in expression across evolved replicated populations (see the “Material and methods” section), we detected pronounced replicate-specific expression changes (Fig. 4a). The heterogeneity in gene expression evolution of reproduction-associated genes was significantly higher than for a random set of genes (Fig. 4b, Wilcoxon’s test, *p* < 0.001). Most of these reproduction-associated genes changed their expression in only one or two replicate population(s) towards “the same” direction (mostly up-regulation) during the adaptation (Fig. 4a and Additional file 7 Fig. S5). Hence, reproduction-related genes are probably under selection in the new environment, but different sets of genes respond in different replicate populations.

## Discussion

The analysis of replicate populations adapting to the same environment demonstrated that reproductive isolation could rapidly evolve during adaptation to a novel temperature regime. The observed patterns of premating reproductive isolation were consistent with the predictions for ecological speciation — populations evolved to different habitats showed reproductive isolation, but no difference was detected between populations adapted to the same environment. Connecting the adaptive response in lipid metabolism of hot-evolved flies to shifts in CHC profiles provides a plausible link between the adaptive response and the pleiotropic byproduct of reproductive isolation. Hence, our study presents one example of incipient ecological adaptation where a likely target of selection (lipid metabolism) has been identified and the pleiotropic effects of involved genes could explain the observed premating reproductive isolation.

The pattern of incipient reproductive isolation among evolved replicate populations is a striking observation. To understand the origins of the postmating reproductive isolation in our study, it is necessary to explain how differences between replicates evolved and why this leads to reproductive isolation. It has been suggested that sexual conflict, driven either by female-male or male-male interactions, could result in replicate specific responses and thus reproductive isolation, but the empirical evidence has remained controversial [36–40]. In addition to selection also genetic drift can cause differences between replicate populations, even if they originate from the same founder population. Therefore, we discuss two alternative hypotheses. First, we explain how uniform selection from standing genetic variation could cause differences between replicates and how this leads to sexual selection and reproductive isolation. Second, we discuss how genetic drift in combination with sexual selection could have triggered postmating reproductive isolation.

It is well-understood that even when the selection pressure operating on a polygenic trait is shared across replicates, it can lead to a heterogeneous genomic [41, 42] and transcriptomic [43] response. This phenomenon is known as genetic redundancy [44, 45]. The observation that reproduction-associated genes (e.g., seminal fluid proteins) showed a higher variation among evolved replicates than other genes (Fig. 4a and b) suggests that alternative combinations of reproduction-associated genes were selected in the different replicates. This pattern fits very well with the redundancy of reproduction-associated proteins [46]—*Drosophila* has, for example, more than 15 trypsin-class proteases in the seminal fluid [47]. We propose that the polygenic adaptation of reproduction-associated proteins in our experiment could have been triggered by the maintenance regime where only females younger than 5 days were able to contribute to the next generation. This led to a dramatic shift towards early fecundity (Hsu et al., unpublished results) and more time and energy investment in mating, as evidenced by a substantially higher chasing activity in evolved males (Fig. 1a). Evidence for the connection between the shift towards early fecundity and reproduction-associated proteins comes from ovulin, a well-studied seminal fluid protein. Ovulin stimulates ovulation by increasing octopamine levels [48] and we showed previously that evolved females have increased octopamine levels [25]. Hence, we assume that the adaptive response towards early fecundity, which is common to all replicates triggered a perturbation of the expression of reproduction-associated genes and their genetic redundancy resulted in the high heterogeneity among replicates.

In a classic polygenic adaptation framework, replicate-specific adaptive responses alone are not sufficient to cause reproductive isolation. Therefore, we propose that an additional evolutionary force is required. For instance, changes in the composition of reproduction-associated genes could result in inter-locus sexual conflict. Sperm must spend some time in the female reproductive tract to fertilize the oocyte, demonstrating that the interaction of male and female components is necessary for successful fertilization [49, 50]. After transfer of sperm and seminal fluid proteins into the female two important processes occur. First, seminal fluid proteins are interacting with proteins of the female reproductive tract. Second, male proteins associated with sperm are being replaced by female peptides [51]. These interactions between the male and female proteins provide an excellent playground for sexual conflict [46]. If an altered composition of reproduction-associated proteins benefits one sex but not the other, conflict arises. Therefore, only (co-evolutionary) changes that are tolerated by both sexes can be favored by selection. Hence, in combination with a polygenic basis of reproduction-associated proteins, replicate populations respond by changes in composition, which are compatible for both sexes within a given population. Crosses between replicates cause reproductive isolation, because incompatible combinations of reproduction-associated proteins are brought together. Evidence from *D. montana* suggests that differences in the composition of seminal fluids are indeed associated with reproductive isolation in natural populations [52]. We propose that adaptation to early fecundity in combination with sexual conflict provides one plausible explanation for the observed postmating reproductive isolation.

It is important to note that similar predictions about the development of postmating reproductive isolation can be made if the expression of reproduction-related genes is not altered by selection (early fecundity), but by genetic drift. A recent theoretical analysis suggested that incompatibilities could arise by genetic drift alone if the allele frequencies of the contributing loci diffuse along a neutral ridge [17]. Since “System Drift” is more likely to generate incompatibilities in small populations than in large ones [18], it is more likely to occur during experimental evolution than in natural *Drosophila* populations. Interestingly, drift-mediated speciation processes have been widely studied and a range of empirical studies demonstrated that reproductive isolation by genetic drift induced by population bottlenecks is unlikely [53–56]. Nevertheless, genetic drift induced by bottlenecks may behave differently than allele frequency changes in moderately sized experimental populations. We think that the continuous operation of genetic drift makes the development of reproductive isolation more likely because it provides the opportunity of co-evolution among the interacting reproduction-related genes. Nevertheless, it is noteworthy that the majority of reproduction-associated genes changed their expression in the same direction (mostly up-regulation) during the experiment. This biased upregulation pattern was specific for reproduction-associated genes and could not be found for other divergently expressed genes (Additional file 8: Fig. S6). It is not clear if such a non-random pattern is expected with system drift. Further work is needed to determine whether system drift results in a higher variance of causal (i.e., reproduction-related) genes among replicates than other genes.

Although our results do not conclusively differentiate between system drift and mutation order speciation, the observed potential for postmating reproductive isolation is noteworthy. How intersexual interactions of the reproduction-related genes contribute to the emergence of incompatibility merits further study. Furthermore, follow-up evolution experiments can be designed to disentangle the relative role of drift and selection for the establishment of reproductive isolation.

## Material and methods

### Experimental evolution

The setup and maintenance of the experimental populations are detailed in [42]. In brief, 10 replicated outbred populations were constituted from the same 202 isofemale lines derived from natural *Drosophila simulans* populations collected in Florida, USA, in 2010. Five females of each line were pooled to create one replicate of a reconstituted ancestral population (henceforth called ancestral population). Replicated populations independently adapted to a laboratory environment at 18/28 °C with 12-h dark/12-h light photoperiod with the census population size of 1250 adult individuals per population per generation. Based on genomic allele frequency changes, an effective population size of ~ 300 was inferred [42]. During the evolution experiment, all replicate populations were reared in the same incubator with randomization orientation/position across generations.

Each founder isofemale line was maintained at a small population size (typically less than 50 individuals) at 18 °C for > 50 generations on standard laboratory food before the reconstitution of the ancestral populations for common garden experiments. Potential adaptation to the lab environment with the residual heterogeneity or de novo mutation is unlikely, as discussed previously [42]. The small effective population size during the maintenance of each line prevents adaptation to the lab environment. This has also been experimentally tested by the lack of significant difference in allele frequencies in populations which were reconstituted shortly after the establishment of the lines and after 50 generations [57]. Furthermore, mutations occurring in the laboratory, if any, are mostly recessive [58], and the effects are likely to be masked because after two generations of random mating during common garden maintenance, most individuals will be heterozygous for isofemale line-specific variants.

## Common garden experiment

We performed multiple common garden experiments with the evolved populations and reconstituted ancestral populations for the transcriptomic, metabolomic, and phenotypic assays in this study. The common garden procedures have been detailed in [25, 26]. Briefly, replicates of ancestral populations were reconstituted by pooling five females each from the founder isofemale lines. These reconstituted ancestral populations and evolved replicates were reared at 18/28 C cycling with a 12-h dark/12-h light photoperiod for at least two generations to minimize transgenerational and/or environmental effects before being subjected to different phenotypic assays.

### Assay for male reproductive activity

We measured the reproductive activity for evolved and reconstituted ancestral populations at generation 140. After four generations at common garden condition (18/28°C cycling), 10 pairs of 5-day-old mated males and females were placed together in an agar-based arena (4% agar, 4% sugar) and filmed for 15 min at 20 FPS (frame-per-second) at 28°C using FlyCapture2 system (PointGrey, Version 2.13.3.31). In total, for matched setup (flies from the same population are assayed together), eight trials within the evolved populations and six trials within the ancestral populations were assayed. In addition, five and six trials of reciprocal mismatched setup (ancestral males were assayed together with evolved females and vice versa) were included in the experiment. Each fly was tracked using a flytracker [59] for movement and behavior analysis. Janelia Automatic Animal Behavior Annotator (JAABA) was used to annotate and recognize the chasing behaviors [60]. The time a male fly spent on chasing females was quantified as its reproductive activity and the difference in the different setups was tested with a Kruskal–Wallis test in *R*.

### Multiple-choice mating assay for assortative mating

We performed a multiple-choice mating assay for assortative mating between (1) three ancestral populations and three selected evolved populations (repl. 1, 3, and 6, generation 194) (2) across the three independently evolved populations. We modified the experimental protocol from [61]. After two generations of common garden condition (18/28 °C cycling with 12-h dark/12-h light photoperiod), 400 unmated males and females were collected from each population in six hours after eclosure. The collected flies were reared on corn meal in vials with moderate density (50 flies per vial, 8 replicates per sex per population) for four days and transferred onto yeast paste saturated with food coloring dyes (red or blue) one day before assaying. During the mating assay, 50 flies of each sex from each population in a combination of interest (one ancestral and one evolved population/two evolved populations) were placed together in a cylindrical cage with a sealable entrance for the aspirator. Eight cages (four for each color) for each population combination were set up and run simultaneously and each cage was checked sequentially. Mating pairs were captured with an aspirator and identified under a microscope based on the color of the abdomen. The examination was terminated when the first 25 mating pairs in a cage were identified or when two hours elapsed. Based on a two-by-two contingency table from the assay for each population combination, Yule’s index (*Y*) [62] was calculated to quantify the strength of assortative mating, and Fisher’s exact test was applied for hypothesis testing.

### Quantification and identification of cuticle hydrocarbons

We measured the cuticle hydrocarbons (CHCs) for five replicates of the ancestral population and 10 independently evolved populations at generation 158. After two generations in a common garden, three replicates of 20 virgin flies of each sex were collected two hours after eclosure from each population. The collected flies were aged for three additional days before CHC extraction. We used 100 µl of heptane containing an internal standard (IS, 10 µg n-C30) to wash the CHCs from each sample. The extracts were stored at – 20 °C until gas chromatography. GC/MS analysis was performed to isolate and quantify each compound. The analysis of chromatograph was performed with an Agilent 7890C gas chromatography system coupled to an Agilent 5975C MSD. Injection volume was 1 µl in an inlet at a temperature of 320 °C, a split of 10:1. He was used as carrier gas at a velocity of 1 ml/min and the compounds separated in a HP-5MS column (30 m × 250 µm ID × 0.25 µm film thickness) with a temperature program 180 °C to 240 °C with 6 C/min and then with 20 C/min to 320 °C. Transfer line temperature to the MSD was 280 °C, the mass range of the MSD was 40–400 m/z, and the electromagnetic voltage was set at 70 eV.

Identification of the constituents was done based on retention index (RI), determined with reference to homologs series of n-alkanes (C8-C30) and mass spectra with the databases NIST 05. Each identified chemical compounds were validated with a published *Drosophila* CHCs chromatograph [29, 34]. For integration, identification, and quantification of compounds, Automated Mass spectral Deconvolution and Identification System (AMDIS) [63] and openChrom [64] were used. The relative concentration of each CHC compound (excluding cis-Vaccenyl acetate) was calculated and subjected to centered-log ratio transformation [63]. Two-way analysis of variance (ANOVA) and principal component analysis (PCA) were performed to dissect the effect of evolution and sex on different CHCs.

### Cross-replicate compatibility assay

At generation 194 of the evolved populations, we performed a diallel cross among three selected evolved replicates (repl. 1, 3, and 6) and measured the number of progenies that survived until adulthood to approximate the fitness of each cross. Since we did not find assortative mating (Fig. 2a), this assay can identify fitness differences caused by viability or fertilization success. The ancestral populations were not included because of the potential impact of assortative mating. After three generations of common garden rearing (18/28°C cycling), 75 unmated males and females were collected from each evolved replicate population and aged to four days old. For each cross, five males and five females from the same/different population were placed together in a vial (with a food surface area of 3.14cm^2^; no crowding effect is expected) at the same common garden condition, and two transfers were made for all vials with a 48-h interval. The number of viable progeny after 14 days from both transfers was averaged and treated as one replicate for the data analysis. Five replicates were generated for each cross and there were in total nine types of cross in a full diallel cross design among three populations. We tested the difference in the number of viable progeny between inter- and intra-population crosses using a linear mixed-effects model to account for a random effect of potential correlation among crosses involving independent flies from the same crossing scheme. The difference in progeny numbers showed marginal statistical significance between intra- and inter-population crosses in a two-tailed test. Given the non-significant random effect (*p* = 0.53), we treated each cross as an independent observation. Subsequently, we conducted a one-tailed Wilcoxon test to test the null hypothesis that intra-population crosses produce an equal or smaller number of progeny compared to inter-population crosses. The significant *p*-value obtained allowed us to reject the null hypothesis.

### RNA-Seq data analysis for divergently expressed genes

RNA-Seq data for the evolution experiment at generation 103 were obtained from previous studies where contrasts between ancestral and evolved populations were made [25, 26]. In this study, we reanalyzed the data for the 5-day-old whole-body male samples of the evolved populations to identify the genes that were divergent across replicates. Only two replicates are available from each evolution replicate for females, preventing similar analysis in females [25, 65]. Genes with count per million (CPM) > 0.1 across all samples were retained and we modeled their expression as $Y=\text{repl}+\varepsilon$, where $Y$ is the normalized expression values; $\text{repl}$ indicates the effect among evolution replicates and $\varepsilon$ is the random error. Likelihood ratio tests implemented in edgeR [66] were used to perform differential expression analysis on the effect of $\text{repl}$. Benjamini and Hochberg’s FDR correction [67] was applied with a FDR cutoff of 0.05. Genes differentially expressed in at least one replicate were identified for further analysis.

### Gene set enrichment analysis

In order to explore the functional implication of the parallel changes and random divergence in the transcriptome, we tested for an enrichment of gene ontology (GO), tissue-specific expression, and reproductive functions among the genes evolving for a parallel response [25] or those evolved for significant divergence across the 10 evolution replicates (this study). GO enrichment was performed using the default “weight01” algorithm implemented in topGO (version 2.32.0) [68]. Genes highly expressed in each tissue were identified based on flyatlas2 expression dataset [69] (required > twofold higher expression in a certain tissue than whole-body). Genes involved in the cuticle hydrocarbon (CHC) metabolism were obtained from [34]. Fisher’s exact tests were applied for the enrichment analysis. Except for the GO enrichment analysis which already accounts for multiple testing [70], Benjamini and Hochberg’s FDR correction [67] was applied to account for multiple testing.

### Replicate-specific evolution in divergently evolving reproductive genes

In order to understand the driving force underlying the divergent evolution in the expression of genes involved in “multicellular organism reproduction” (GO:0032504), we investigated the replicate-specific gene expression evolution of 255 reproductive genes in this term that were significantly diverged across independent replicates. Using the expression matrix of these genes in all samples (Additional file 4: Fig. S4), we summarized the variation in expression of the 255 genes by the principal components (PCs). The PC scores of each sample reflect the expression difference across the sample. Thus, comparing different evolved replicates to the ancestral samples could inform us about replicate-specific evolution in a certain subset of genes. Additionally, we investigated the heterogeneity in the expression changes across replicates as:

$$\text{Heterogeneity}=\sum_{i,j}\left(1-\text{cor}\left({x}_{i},{x}_{j}\right)\right)/N$$

where *x* is a vector consisting of the expression change of each gene in each evolution replicate, $i,j\in \left[\text{1,10}\right], i\ne j$ and *N* is the total number of pairwise combinations of 10 replicates.

### Supplementary Information

Additional file 1: Fig. S1. Gas chromatography of the cuticular hydrocarbons (CHCs) in both sexes of Drosophila simulans. Fig. S2. Expression evolution of genes associated with cuticular hydrocarbons (CHCs) metabolism in both sexes of Drosophila simulans. Fig. S3. Evolution of post-mating incompatibility among independently evolved populations. Fig. S4. Transcriptomic divergence among replicate populations adapting to the same environment. Fig. S5. Transcriptomic divergence of reproduction-related genes among replicate populations. Fig. S6. The predominance of up-regulation is specific for reproduction-related genes.

Additional file 2: Table S1. Genes significantly diverged across evolutionary replicates. edgeR output table for the test among evolutionary replicates.

Additional file 3: Table S2. Gene ontology (GO) enrichment analysis for the significantly diverged across evolutionary replicates. topGO output table with the weight01 algorithm for 3,062 significantly diverged genes among evolutionary replicates.

Additional file 4.

## Data Availability

Sequence reads from this study are available from the European Sequence Read Archive (http://www.ebi.ac.uk/ena/) under the study accession number PRJEB35504 [71] and PRJEB35506 [72]. Additional scripts and raw data are available on Github [73] and Zenodo [74] under an MIT license.
