# Supplementary material for: Reproductive isolation arises during laboratory adaptation to a novel hot environment
Source: Genome Biol. 2024 May 28;25:141. doi: 10.1186/s13059-024-03285-9 (PMC11134630; doi:10.1186/s13059-024-03285-9)
Supplement: Supplementary file 1 — Additional file 1: Fig. S1. Gas chromatography of the cuticular hydrocarbons (CHCs) in both sexes of Drosophila simulans. Fig. S2. Expression evolution of genes associated with cuticular hydrocarbons (CHCs) metabolism in both sexes of Drosophila simulans. Fig. S3. Evolution of post-mating incompatibility among independently evolved populations. Fig. S4. Transcriptomic divergence among replicate populations adapting to the same environment. Fig. S5. Transcriptomic divergence of reproduction-related genes among replicate populations. Fig. S6. The predominance of up-regulation is specific for reproduction-related genes. [file 13059_2024_3285_MOESM1_ESM.docx]

Supplementary Information for

Pre- and postmating reproductive isolation arises during adaptation to a novel hot environment

Sheng-Kai Hsu, Wei-Yun Lai, Johannes Novak, Felix Lehner, Ana Marija Jakšić, Elisabetta Versace, Christian Schlötterer

Correspondence to Christian Schlötterer

Email: christian.schloetterer@vetmeduni.ac.at

**This PDF file includes:**

Fig. S1 to S6

**Other supplementary materials for this manuscript include the following:**

Additional file 5:
**Table S1. Genes significantly diverged across evolutionary replicates.**edgeR output table for the test among evolutionary replicates.

Additional file 6:

**Table S2. Gene ontology (GO) enrichment analysis for the significantly diverged across evolutionary replicates.**

topGO output table with the weight01 algorithm for 3,062 significantly diverged genes among evolutionary replicates.


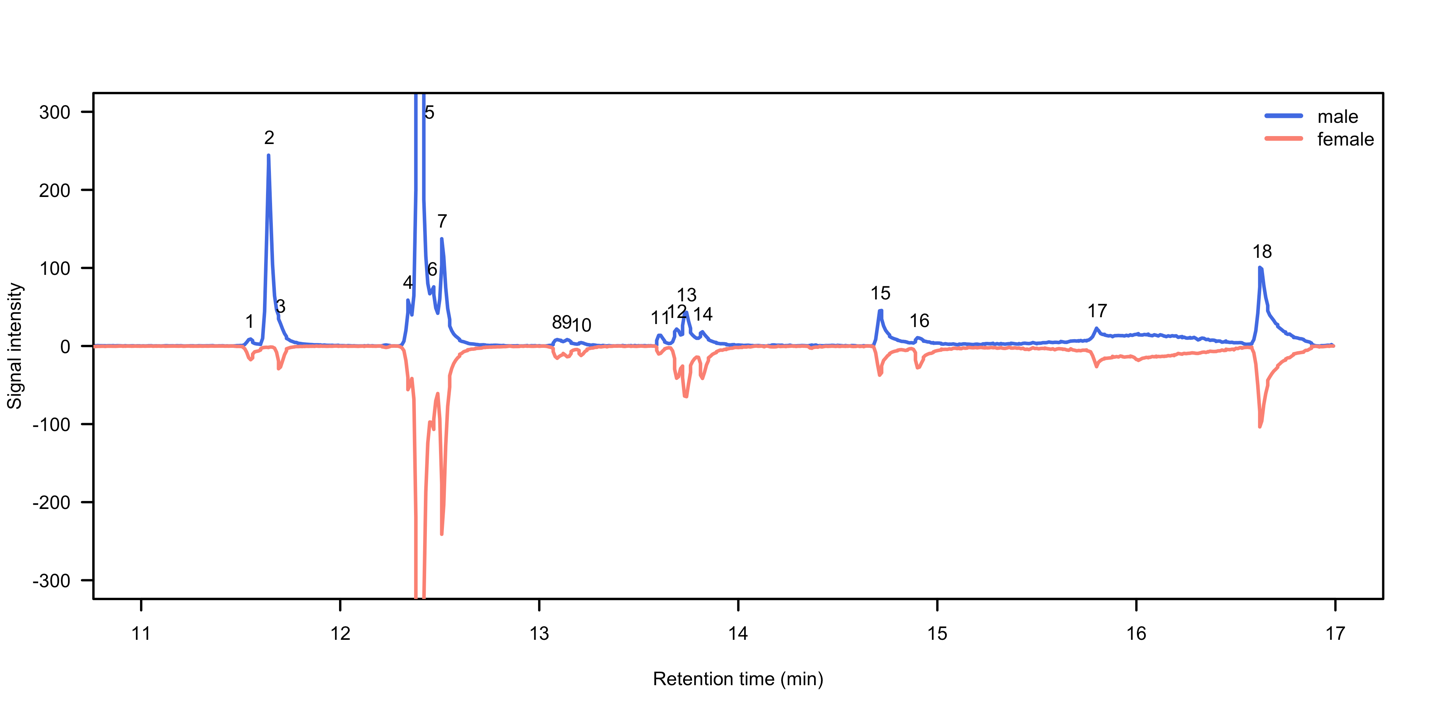


**Fig. S1. Gas chromatography of the cuticular hydrocarbons (CHCs) in both sexes of *Drosophila simulans*.** The x axis denotes the retention time and the y axis indicates the signal intensity. Blue curve is the chromatography for male and red curve is for female. In total, 18 peaks can be detected. The major sex difference is the presence and absence of peak 2 which is identified as cis-vaccenyl acetate (c-VA).


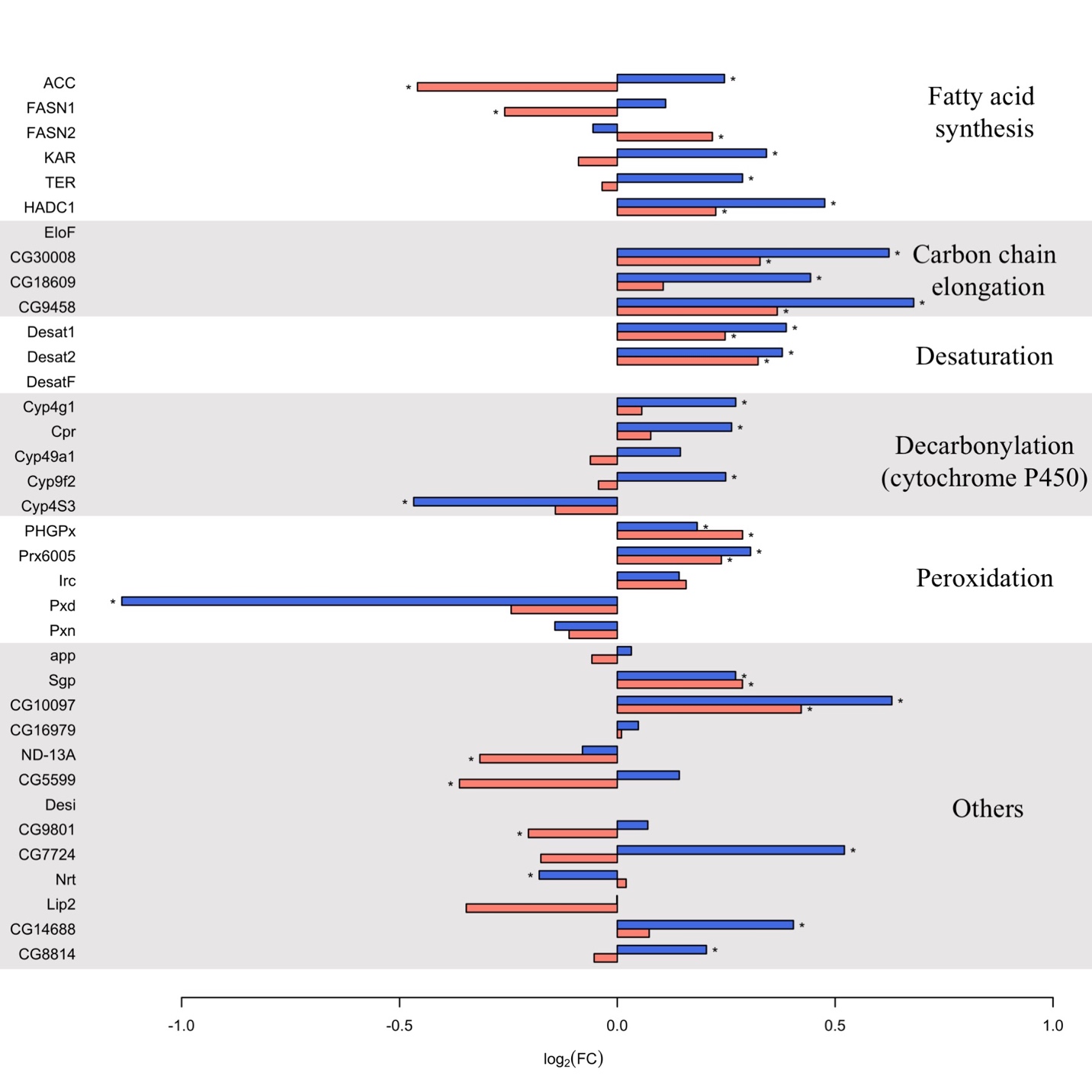


**Fig. S2. Expression evolution of genes associated with cuticular hydrocarbons (CHCs) metabolism in both sexes of *Drosophila simulans*.** A substantial number of genes reported to be associated with CHC metabolism (Dembeck et al., 2015) evolved for expression changes during the adaptation to a novel hot environment. The expression changes are represented by log_2_-fold change before and after the adaptation. Red bars denote female changes and blue bars for male. The asterisks indicate statistical significance (FDR < 0.05).


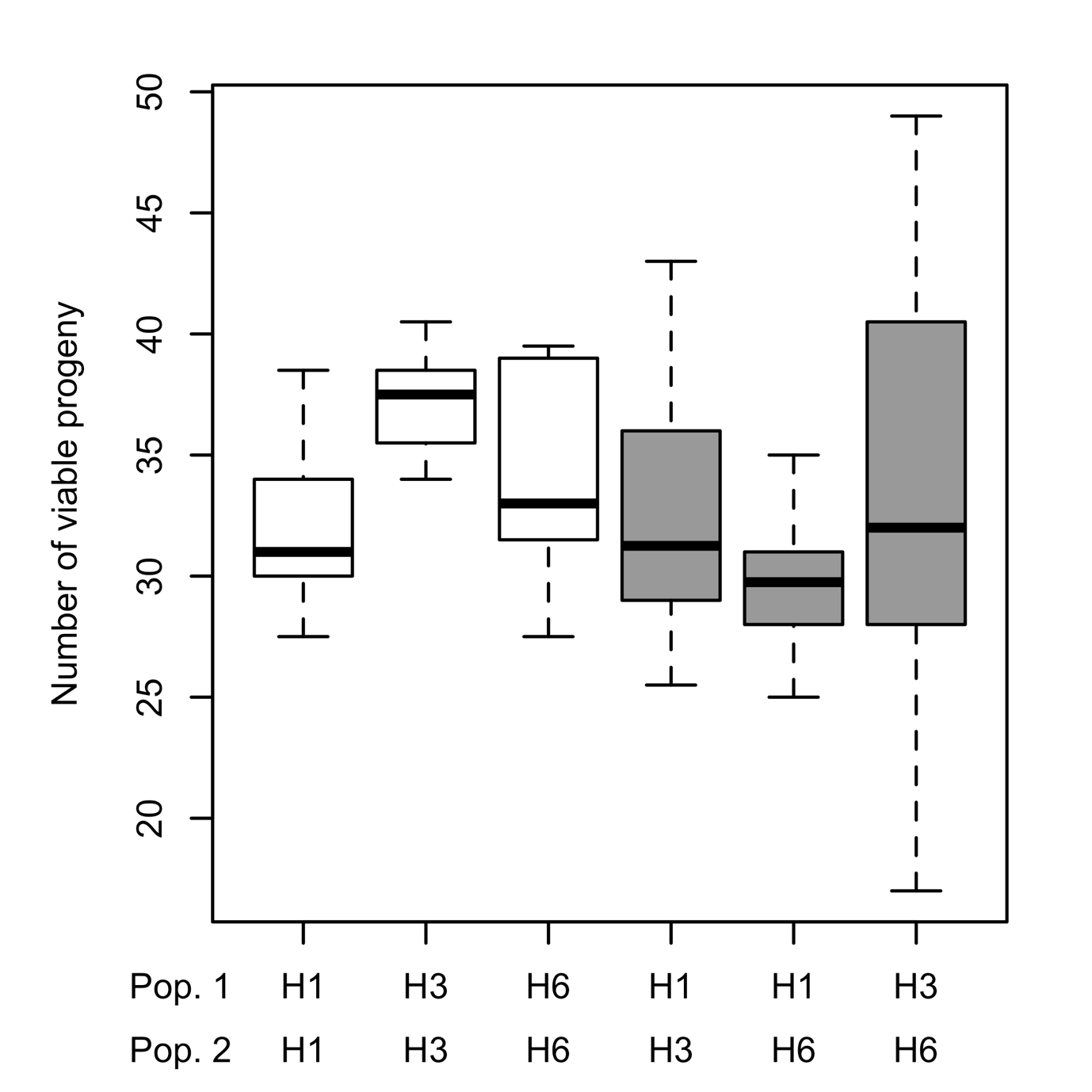


**Fig. S3. Evolution of post-mating incompatibility among independently evolved populations.** Fitness measures of different crosses between replicate populations. The fitness was measured as the numbers of viable adults in each cross. White boxes denote con-population crosses while grey boxes indicate hetero-population crosses. The fitness of hetero-population (between replicates) crosses was always lower than the mid-parent fitness.


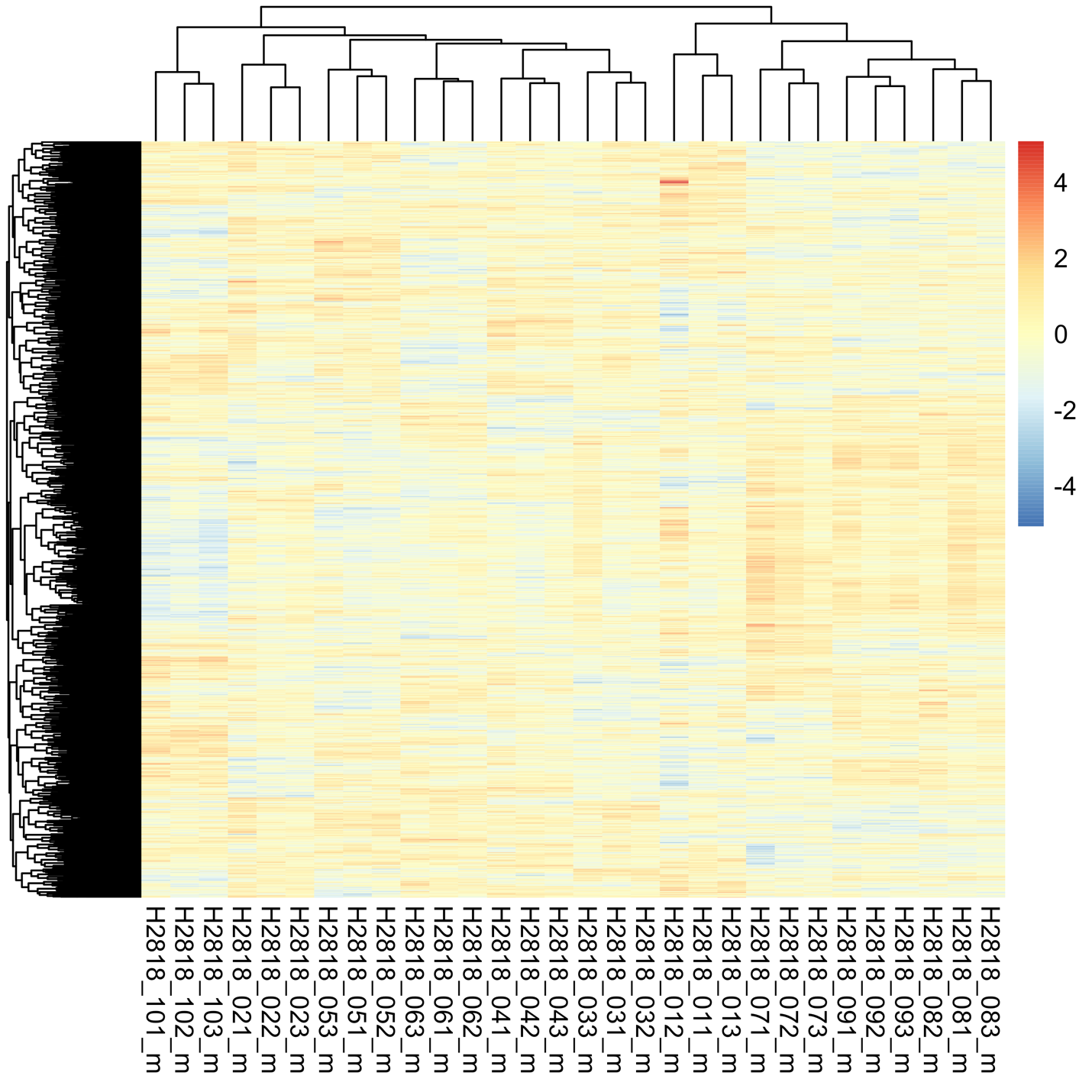


**Fig. S4. Transcriptomic divergence among replicate populations adapting to the same environment.** Normalized expression values of the 3,062 genes exhibiting significant expression changes across replicates are shown (heat color in each cell). Each row indicates a gene and each column is one biological samples. Based on the expression of these divergently expressed genes, the samples from the same replicate populations are clustered together.


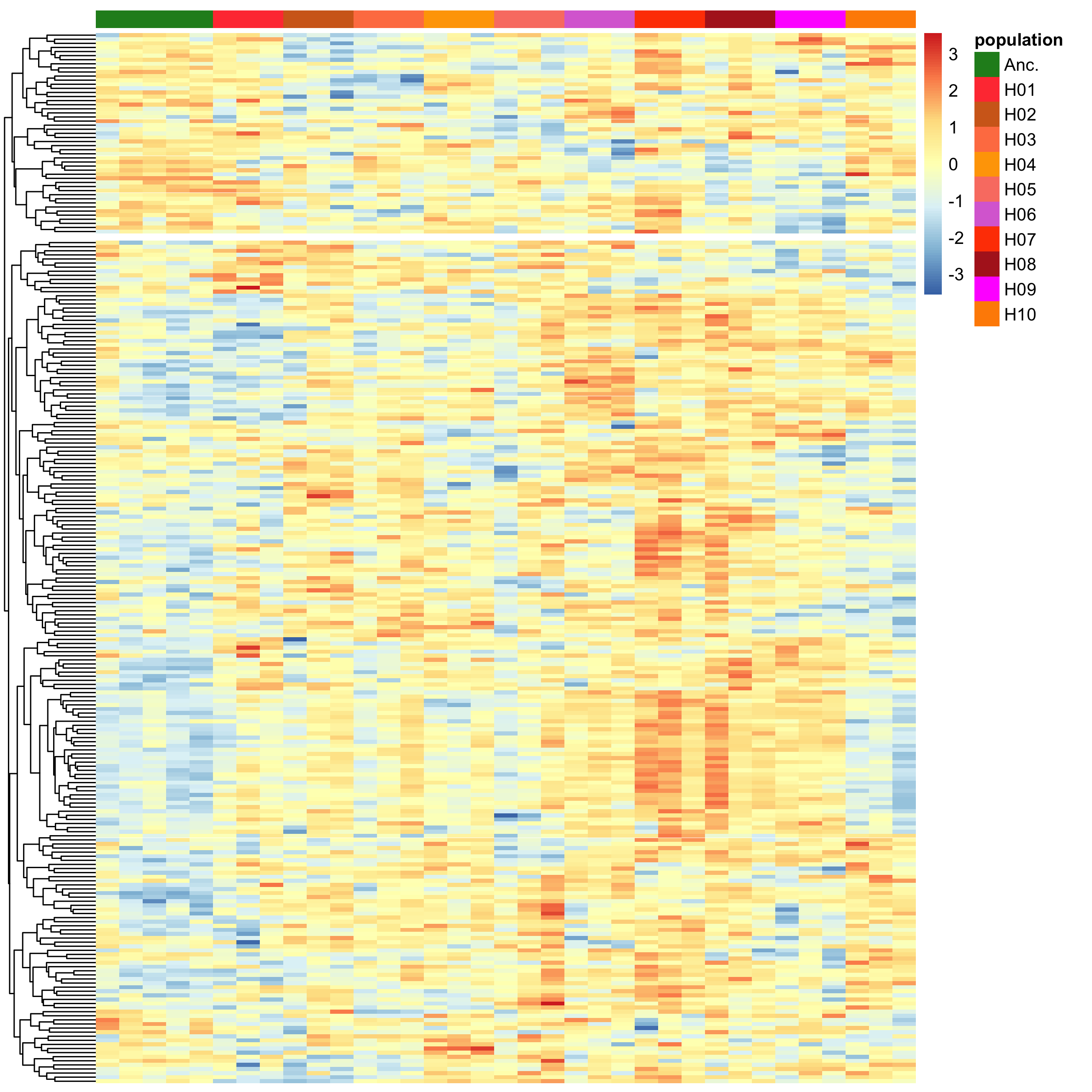


**Fig. S5. Transcriptomic divergence of reproduction-related genes among replicate populations.** Normalized expression values of the 255 reproduction-related genes exhibiting significant expression divergence across replicates are shown (heat color in each cell). Each row indicates a gene and each column is one biological samples. The column annotation indicates the identities of each biological samples. Most of the reproduction-related genes evolved for up-regulation but distinct sets of genes evolved in different replicates.


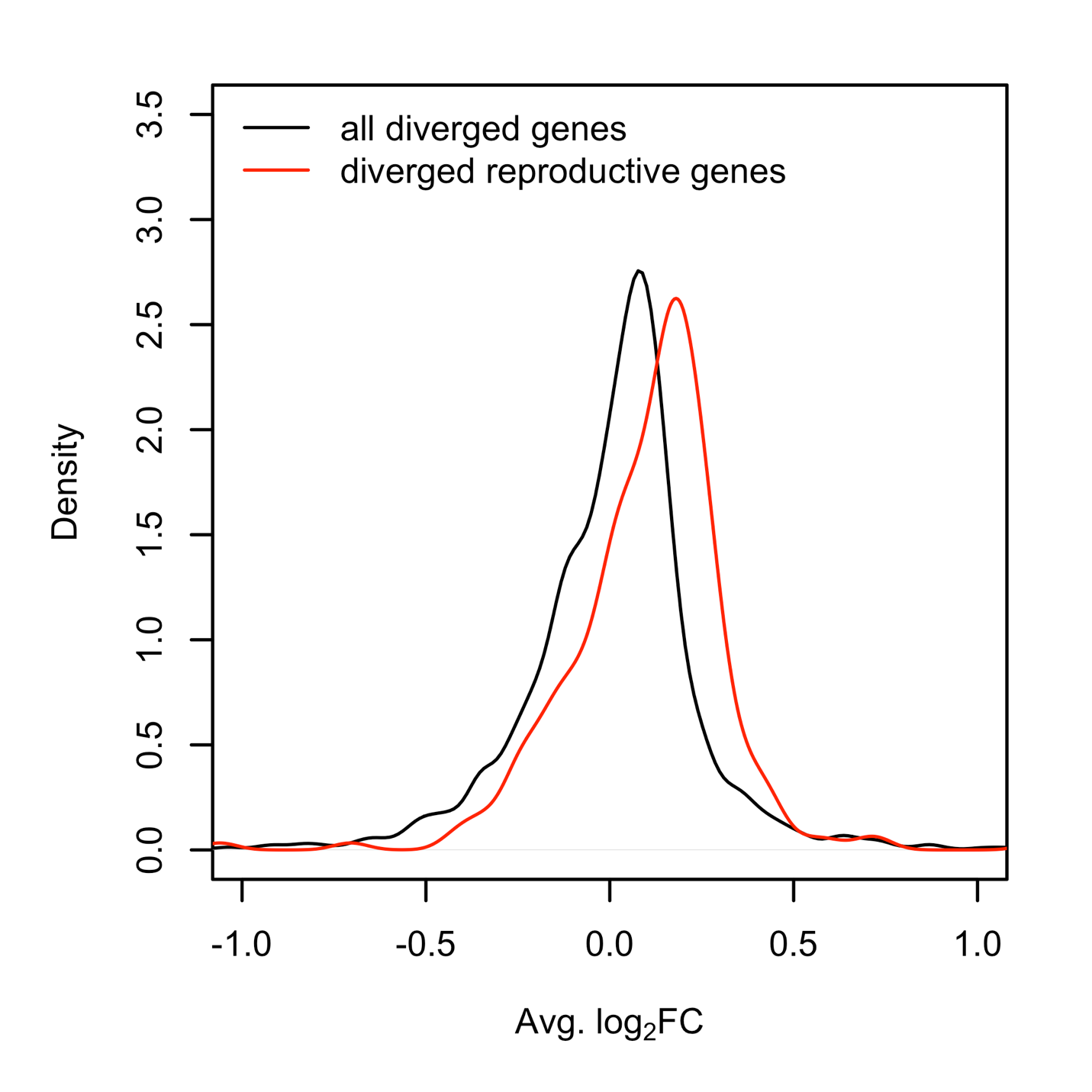


**Fig. S6. The predominance of up-regulation is specific for reproduction-related genes.** Average expression difference between the ancestral and evolved populations (log_2_-scaled fold change) for each of the 255 reproduction-associated genes were compared to all divergently expressed genes. The systematic shift toward higher expression is statistically significant (Wilcoxon’s test, p < 0.001).
