# Supplementary material for: Reproductive isolation arises during laboratory adaptation to a novel hot environment
Source: Genome Biol. 2024 May 28;25:141. doi: 10.1186/s13059-024-03285-9 (PMC11134630; doi:10.1186/s13059-024-03285-9)
Supplement: Supplementary file 4 — Additional file 4. [file 13059_2024_3285_MOESM4_ESM.docx]

**Review history**

**First round of review**

**Reviewer 1**

Hsu et al. present evidence for two different mechanism of speciation occurring in Drosophila simulans populations adapting to new environmental conditions in the lab. First, they find that replicate evolved populations show pre-mating reproductive isolation to a reconstructed ancestral population, but not to other populations that evolved in the same environment. This is consistent with ecological speciation. Second, they find evidence of post-mating reproductive isolation between replicate populations that evolved in the same environment. This is consistent with mutation-order speciation. By measuring cuticular hydrocarbon (CHC) composition and transcript abundance in the populations, they attribute the first observation to adaptive differences in lipid metabolism and the second observation to differences in the expression of reproduction-related genes. The specific results present in this manuscript are clear and supported by appropriate statistical analyses. They present original and convincing data. However, I am not convinced by their interpretation of the data or, more generally, their claims regarding speciation, which is the major focus of the manuscript.

1) The authors focus on "speciation", but I'm not sure they can make conclusions about speciation from their results. Adding "incipient" in front of "speciation" is not really a solution in this case. As these authors and others have shown, polygenic adaptation may lead to subtle frequency changes at many loci, not fixed differences between populations. Thus, although there may be phenotypic differentiation and reproductive isolation, there may not be sufficient genetic divergence to cause speciation. With minor differences in allele frequency and weak reproductive isolation between populations (as is observed here), it is not obvious that the proposed mechanisms would lead to speciation. Gene flow between populations could quickly homogenise them. Additional mechanisms would be needed to generate true, incompatible species.

2) Are the authors suggesting that the populations are continuing to adapt after 140 generations and that CHC and reproductive traits will continue to diverge? Or did polygenic adaptation allow the populations to quickly reach a new phenotypic optimum? They should have some data regarding phenotypes and allele trajectories over the course of the laboratory evolution experiment to address this question. If traits have already reached a new optimum, then it is unlikely that continued evolution will lead to speciation, at least not adaptive speciation.

3) Given the two points mentioned above, it would be more appropriate if the authors tone down their claims regarding speciation and re-formulate the manuscript in terms of "reproductive isolation" or, perhaps better, "partial reproductive isolation". The discussion could then be expanded to cover the issues raised above and whether the observed partial reproductive isolation may (or may not) represent an early stage in speciation.

4) Postzygotic isolation is inferred from progeny number and attributed to reproductive genes, but if one is only counting adult offspring, there is no way to know if it is a reproductive incompatibility or something else affecting development or viability. Reproductive genes are known to evolve rapidly at the sequence and expression level, which is what the authors see here. However, it is not shown that this rapid evolution is the cause of postzygotic reproductive isolation. Maybe the argument could be strengthened if they had counted eggs, larvae, pupae, and adults resulting from their crosses. Then one could determine the stage at which the isolation occurs. As I understand, they only looked at eclosed adults in the next generation? This includes differences in fecundity, and well as viability. In the end, the link between reproductive gene expression and the observed postzygotic isolation is rather tenuous.

5) There are some places in the manuscript where I think "progenies" should be "progeny" (l. 332, Y-axis of Fig 4b).

**Reviewer 2**

This is an interesting study on the potential evolution of partial reproductive isolation associated with experimental evolution in a challenging environment. However, the present manuscript has several substantial flaws and considerable revision would be needed to reach publishability.

Positives:
* The idea of looking at reproductive isolation among experimentally evolved populations is very promising and I have wondered if/when the lab would turn to that topic.
* The evidence for (partial) pre-mating isolation, in terms of male courtship preference, is clear, and it does have some general relevance to the ecological speciation model.
* The integration of gene expression analyses with reproduction-oriented experiments provides complementary perspectives.
* The text is mostly clear and well-written, with some exceptions noted.

Negatives:
* The evidence for (partial) post-mating isolation is rather limited.
* To the extent that post-mating isolation exists, the authors fail to adequately consider the potential for genetic drift to underlie it.
* The narrow framing of the study leads to notably poor scholarship. For example, there is basically no acknowledgment of previous studies on laboratory evolution and reproductive isolation.
* There is a fair amount of over-interpretation and over-simplification in this manuscript.

Out of typical expediency, I focus below exclusively on the areas where improvement is needed.


Major points:

1. The authors assume that only natural selection can lead to reproductive isolation. They begin the Introduction by presenting a highly debatable claim as if it were a universally-acknowledged fact: "Most new species are formed by selection [1-3] as already suggested by Darwin [4]." Then, they proceed to frame their study strictly in terms of two specific models of selection-driven reproductive isolation: ecological speciation and mutation-order speciation. Another alternative the authors really must acknowledge is genetic drift. Drift makes the same prediction as mutation-order speciation (i.e. that reproductive isolation may occur between any populations, regardless of environment). The authors previously estimated that their experimental populations had an effective population size of around 300. The experiments reported here were conducted at various generations, between 100 and 200 generations after the lab populations were assembled. Hence, we are talking about roughly 0.3-0.7Ne generations, which implies powerful drift (in addition to powerful selection). Schiffman & Ralph (2022) described conditions in which incompatibilities could arise neutrally in a scenario such as this. Quoting from their abstract: "system drift, is expected to proceed at a rate proportional to the amount of intrapopulation genetic variation divided by the effective population size (⁠Ne⁠). At biologically reasonable parameter values this could lead to substantial interpopulation incompatibility, and thus speciation, on a time scale of Ne generations.". In these experiments, there is a ton of variation going into the heat-exposed populations (from a highly diverse natural fly population), and yet lab Ne is small, creating a large ratio of variation to Ne, which entails far more favorable conditions for the system drift model than would exist in a constant-size natural population. Unfortunately, the authors do not seem to have any outbred control populations subject to drift but not thermal selection, which might otherwise be useful in testing between the drift and mutation order hypotheses (although they would still experience lab adaptation).

The authors only belatedly mention drift in the latter part of their brief Discussion section. Their reasons for dismissing it revolve entirely around the expression patterns of reproductive genes in heat-evolted populations. However, they can not prove that the evolution of reproductive genes is related to the tentative signal of post-mating isolation they report (in other words, the expression patterns could be non-neutral but not contribute to post-mating isolation). Alternatively, even some of the evolution of reproductive genes could reflect drift, due to the reduced effectiveness of selection on sex-specific traits (Dapper & Wade 2020).

In the absence of any clearer evidence to differentiate these hypotheses, system drift must be placed on at least equal footing with mutation order speciation, including in the abstract.

2. The current manuscript is completely bereft of relevant context from prior laboratory evolution studies. There is an entire branch of literature on the evolution of reproductive isolation in laboratory settings that is totally missing from this manuscript. As a starting point, I have copied several links at the bottom of my review, but there are almost certainly other relevant references out there as well. Such background must be summarized in the Introduction for the manuscript to reach a publishable state. Other areas of the literature are under-cited as well, including the roles of CHCs in Drosophila ecological adaptation and reproductive isolation.

3. The Results arguing for postmating isolation are limited and not especially convincing. The authors look at the numbers of F1 progeny from crosses within vs. between the 3 heat-selected replicate populations. They show only a coarse summary of these results (they should be presented in more detail in a supplemental table), which entail a modestly (8%) lower reproductive rate for the between-population crosses. The authors report a modestly significant P value (0.03) for this difference, although given the diallel design and the 5 replicates per cross, I'm not sure the assumptions of the Wilcoxon test are met. There is also ambiguity about what was actually done. This statement from the Results makes it sound like they looked at the offspring of F1s instead...
"F1 flies from crosses between replicates of the evolved 148 populations produced 8.3% fewer viable offspring"
...but the Methods text implies that they instead looked at F1 offspring of the crossed parental populations. Assuming the latter is correct, I am puzzled the authors did not look at the viability or reproductive success of F2 offspring, which are much more likely to manifest incompatibilities than F1s.

4. Related to the above, there is a lot of simplistic and over-confident interpretation throughout the manuscript. Examples:

Abstract: "Gene expression analysis identified the underlying molecular mechanisms"
Over-statement - no causal demonstration of mechanism is reported.

Abstract: "Premating ecological speciation is the byproduct of an altered lipid metabolism"
Falsely implies that speciation has fully occurred here.

Intro: "We find support for ecological speciation"
As with the abstract, some nuance is needed here to avoid suggesting that complete speciation has occurred. "support for the ecological speciation model" would be more appropriate.

Disc: "the pleiotropic effects of involved genes explain the premating reproductive isolation"
This is a promising hypothesis but it was not formally demonstrated here.

Disc: "Remarkably, we did not only find evidence for ecological speciation, but also for mutation order speciation in the same experiment"
Same issues as above.

And throughout the manuscript, the term "reproductive isolation" is used rather loosely. It might be interpreted as meaning "these fly populations are now reproductively isolated", when in fact the claim is just that there is a non-zero level of pre-mating and post-mating isolation between them. More careful language is called for.


Specific things:

I agree with the authors that mutation-order speciation is conceptualized too narrowly and that standing variation and multiple loci is a more broadly relevant scenario. An alternative term that more generally invokes "contingency" in adaptation might be an improvement to the terminology of the field.

Figure 1 could be a starting point for a graphical abstract, but it doesn't really add much as a main paper figure.

Figure 2A's axis labels and legend description are vaguely/ambiguously presented. Please clarify the males and females involved in each experiment. It's also not clear why there need to be "post-hoc groups". In Figure 2B, while I can guess what A and H might refer to, they aren't defined in the legend. The nature of the experiment and the conceptual meaning of the metric should also be spelled out in the legend.

Data Availability should be improved at this stage:
"Additional scripts and raw data are available on Github upon publication."
This is not a publishable statement. The authors need to clearly present what they intend to share publicly, so that reviewers can determine whether it is sufficient.


Small changes:

Line 52: "another" -> "other"

Line 87: "drives" -> "may contribute to"

Line 95: "females" -> "hot-evolved females"

Line 96: "same pattern"
Spell out more clearly that ancestral males spent less time courting hot-evolved females than ancestral females, assuming this is what is meant here.

Line 122: "indicating parallel evolution of CHC composition in both sexes"
"either pleiotropic or parallel evolution" would seem more appropriate. Parallel evolution implies genetically separate changes for each sex, and it's not clear if that is true.

Line 137: "Desaturases" and "elongases" should not be italicized.\

Line 140: "can explain" -> "may contribute to"

Line 156: From females, males, or both?

Line 243: missing period

Line 350: "Only two replicates are available from each evolution replicates for females"
Clarify double usage of "replicates".


https://academic.oup.com/evolut/article-abstract/57/11/2557/6756995
https://link.springer.com/article/10.1007/BF02003975
https://academic.oup.com/evolut/article-abstract/69/12/3141/6852102
https://www.pnas.org/doi/abs/10.1073/pnas.1901247116
https://onlinelibrary.wiley.com/doi/full/10.1111/jeb.12246
https://journals.plos.org/plosgenetics/article?id=10.1371/journal.pgen.1002056
https://onlinelibrary.wiley.com/doi/full/10.1002/ece3.413
https://www.researchgate.net/profile/James-Fry/publication/265181361_Laboratory_Experiments_on_Speciation/links/5512ebe00cf268a4aaeb8a2b/Laboratory-Experiments-on-Speciation.pdf

**Authors’ response to reviewers**

Reviewer reports:

Reviewer #1: Hsu et al. present evidence for two different mechanism of speciation occurring in Drosophila simulans populations adapting to new environmental conditions in the lab. First, they find that replicate evolved populations show pre-mating reproductive isolation to a reconstructed ancestral population, but not to other populations that evolved in the same environment. This is consistent with ecological speciation. Second, they find evidence of post-mating reproductive isolation between replicate populations that evolved in the same environment. This is consistent with mutation-order speciation. By measuring cuticular hydrocarbon (CHC) composition and transcript abundance in the populations, they attribute the first observation to adaptive differences in lipid metabolism and the second observation to differences in the expression of reproduction-related genes. The specific results present in this manuscript are clear and supported by appropriate statistical analyses.
They present original and convincing data. However, I am not convinced by their interpretation of the data or, more generally, their claims regarding speciation, which is the major focus of the manuscript.

1) The authors focus on "speciation", but I'm not sure they can make conclusions about speciation from their results. Adding "incipient" in front of "speciation" is not really a solution in this case. As these authors and others have shown, polygenic adaptation may lead to subtle frequency changes at many loci, not fixed differences between populations. Thus, although there may be phenotypic differentiation and reproductive isolation, there may not be sufficient genetic divergence to cause speciation. With minor differences in allele frequency and weak reproductive isolation between populations (as is observed here), it is not obvious that the proposed mechanisms would lead to speciation. Gene flow between populations could quickly homogenise them. Additional mechanisms would be needed to generate true, incompatible species.

The reviewer takes a very extreme position regarding the use of speciation, which is not even met for taxonomically recognized species-in particular for plants. For good reasons several speciation concepts relax this assumption of no gene flow. Hence, we think that dismissing the term incipient speciation is a very extreme position, which is not shared by the Drosophila community. Below we provide some examples of publications that use incipient speciation in situations that closely match the one of our study.

Nevertheless, we do not want to imply that no additional mechanisms are needed to make the transition from incipient speciation (reproductive isolation) to “proper” species with complete reproductive isolation. It is a well-known problem of speciation research that the analysis of well-separated species cannot distinguish between primary and secondary (and tertiary) effects. For this reason, research on incipient speciation is widely considered a good approach to study speciation processes-with the obvious limitation mentioned that it is not clear whether this incipient speciation will ultimately be completed. In any case, this critique applies to an entire research field and should not be used as evidence against our manuscript.

1. Incipient speciation in Drosophila melanogaster involves chemical signals.

Grillet M, Everaerts C, Houot B, Ritchie MG, Cobb M, Ferveur JF.

Sci Rep. 2012;2:224. doi: 10.1038/srep00224. Epub 2012 Jan 19.

PMID: 22355738 Free PMC article.

2. Drosophila yakuba mayottensis, a new model for the study of incipient ecological speciation.

Yassin A.

Fly (Austin). 2017 Jan 2;11(1):37-45. doi: 10.1080/19336934.2016.1221550. Epub 2016 Aug 11.

PMID: 27560369 Free PMC article.

3. Genetics of incipient speciation in Drosophila mojavensis. III. Life-history divergence in allopatry and reproductive isolation.

Etges WJ, De Oliveira CC, Noor MA, Ritchie MG.

Evolution. 2010 Dec;64(12):3549-69. doi: 10.1111/j.1558-5646.2010.01096.x.

PMID: 20681983

4. Incipient speciation by sexual isolation in Drosophila: concurrent evolution at multiple loci.

Ting CT, Takahashi A, Wu CI.

Proc Natl Acad Sci U S A. 2001 Jun 5;98(12):6709-13. doi: 10.1073/pnas.121418898.

PMID: 11390997 Free PMC article.

5. Some evidence of incipient speciation in Drosophila kikkawai.

Gupta JP, Sundaran AK.

Genome. 1994 Dec;37(6):1041-4. doi: 10.1139/g94-148.

PMID: 7828836

6. Drosophila flies in "Evolution Canyon" as a model for incipient sympatric speciation.

Korol A, Rashkovetsky E, Iliadi K, Nevo E.

Proc Natl Acad Sci U S A. 2006 Nov 28;103(48):18184-9. doi: 10.1073/pnas.0608777103. Epub 2006 Nov 15.

PMID: 17108081 Free PMC article.

7. INCIPIENT SPECIATION BY SEXUAL ISOLATION IN DROSOPHILA MELANOGASTER: VARIATION IN MATING PREFERENCE AND CORRELATION BETWEEN SEXES.

Hollocher H, Ting CT, Pollack F, Wu CI.

Evolution. 1997 Aug;51(4):1175-1181. doi: 10.1111/j.1558-5646.1997.tb03965.x.

PMID: 28565503

8. Genetics of incipient speciation in Drosophila mojavensis: II. Host plants and mating status influence cuticular hydrocarbon QTL expression and G x E interactions.

Etges WJ, de Oliveira CC, Ritchie MG, Noor MA.

Evolution. 2009 Jul;63(7):1712-30. doi: 10.1111/j.1558-5646.2009.00661.x. Epub 2009 Feb 18.

PMID: 19228188

9. Experimental Introgression To Evaluate the Impact of Sex Specific Traits on Drosophila melanogaster Incipient Speciation.

Cortot J, Farine JP, Houot B, Everaerts C, Ferveur JF.

G3 (Bethesda). 2019 Aug 8;9(8):2561-2572. doi: 10.1534/g3.119.400385.

PMID: 31167833 Free PMC article.

10. Genetics of incipient speciation in Drosophila mojavensis. I. Male courtship song, mating success, and genotype x environment interactions.

Etges WJ, de Oliveira CC, Gragg E, Ortíz-Barrientos D, Noor MA, Ritchie MG.

Evolution. 2007 May;61(5):1106-19. doi: 10.1111/j.1558-5646.2007.00104.x.

PMID: 17492965

11. Incipient speciation by sexual isolation in Drosophila melanogaster: extensive genetic divergence without reinforcement.

Hollocher H, Ting CT, Wu ML, Wu CI.

Genetics. 1997 Nov;147(3):1191-201. doi: 10.1093/genetics/147.3.1191.

PMID: 9383062 Free PMC article.

12. MORPHOMETRIC EVIDENCE FOR INCIPIENT SPECIATION IN DROSOPHILA SILVESTRIS FROM THE ISLAND OF HAWAII.

Carson HL, Val FC, Simon CM, Archie JW.

Evolution. 1982 Jan;36(1):132-140. doi: 10.1111/j.1558-5646.1982.tb05018.x.

PMID: 28581101 No abstract available.

13. Genetic evidence for adaptation-driven incipient speciation of Drosophila melanogaster along a microclimatic contrast in "Evolution Canyon," Israel.

Michalak P, Minkov I, Helin A, Lerman DN, Bettencourt BR, Feder ME, Korol AB, Nevo E.

Proc Natl Acad Sci U S A. 2001 Nov 6;98(23):13195-200. doi: 10.1073/pnas.231478298. Epub 2001 Oct 30.

PMID: 11687637 Free PMC article.

14. Sexual isolation in Drosophila melanogaster: a possible case of incipient speciation.

Wu CI, Hollocher H, Begun DJ, Aquadro CF, Xu Y, Wu ML.

Proc Natl Acad Sci U S A. 1995 Mar 28;92(7):2519-23. doi: 10.1073/pnas.92.7.2519.

PMID: 7708677 Free PMC article.

15. Female preferences between incipient species of Drosophila athabasca reveal divergent closed functions, epistasis, and segregation of veiled preference alleles.

Yukilevich R.

Evolution. 2023 Feb 4;77(2):496-508. doi: 10.1093/evolut/qpac055.

PMID: 36626802

16. Change in a secondary sexual character as evidence of incipient speciation in Drosophila silvestris.

Carson HL, Bryant PJ.

Proc Natl Acad Sci U S A. 1979 Apr;76(4):1929-32. doi: 10.1073/pnas.76.4.1929.

PMID: 16592642 Free PMC article.

17. Genetic differentiation and adaptive evolution at reproductive loci in incipient Drosophila species.

Almeida FC, DeSalle R.

J Evol Biol. 2017 Mar;30(3):524-537. doi: 10.1111/jeb.13021. Epub 2016 Dec 19.

PMID: 27883252

18. Ecological adaptation during incipient speciation revealed by precise gene replacement.

Greenberg AJ, Moran JR, Coyne JA, Wu CI.

Science. 2003 Dec 5;302(5651):1754-7. doi: 10.1126/science.1090432.

PMID: 14657496

19. Adaptive loss of an old duplicated gene during incipient speciation.

Greenberg AJ, Moran JR, Fang S, Wu CI.

Mol Biol Evol. 2006 Feb;23(2):401-10. doi: 10.1093/molbev/msj045. Epub 2005 Oct 26.

PMID: 16251509

2) Are the authors suggesting that the populations are continuing to adapt after 140 generations and that CHC and reproductive traits will continue to diverge? Or did polygenic adaptation allow the populations to quickly reach a new phenotypic optimum? They should have some data regarding phenotypes and allele trajectories over the course of the laboratory evolution experiment to address this question. If traits have already reached a new optimum, then it is unlikely that continued evolution will lead to speciation, at least not adaptive speciation.

We do not have data on the time-dynamics of adaptation, but we think that the new trait optimum has been reached. Nevertheless, we think that our data are fully in line with previous studies in Drosophila, which also talk about incipient speciation, although full reproductive isolation has not been shown.

3) Given the two points mentioned above, it would be more appropriate if the authors tone down their claims regarding speciation and re-formulate the manuscript in terms of "reproductive isolation" or, perhaps better, "partial reproductive isolation". The discussion could then be expanded to cover the issues raised above and whether the observed partial reproductive isolation may (or may not) represent an early stage in speciation.

In response to the concern of the reviewer, we have changed the wording of our manuscript. We only talk about reproductive isolation, which matches the predicted patterns of some speciation processes. The discussion has been expanded to explain the assumed mechanisms in more detail.

4) Postzygotic isolation is inferred from progeny number and attributed to reproductive genes, but if one is only counting adult offspring, there is no way to know if it is a reproductive incompatibility or something else affecting development or viability. Reproductive genes are known to evolve rapidly at the sequence and expression level, which is what the authors see here. However, it is not shown that this rapid evolution is the cause of postzygotic reproductive isolation. Maybe the argument could be strengthened if they had counted eggs, larvae, pupae, and adults resulting from their crosses. Then one could determine the stage at which the isolation occurs. As I understand, they only looked at eclosed adults in the next generation? This includes differences in fecundity, and well as viability. In the end, the link between reproductive gene expression and the observed postzygotic isolation is rather tenuous.

Our focus has been on the pattern of reproductive isolation that matches the predictions of mutation order speciation: more offspring in conspecific than heterospecific crosses. Hence, we did not further dissect the developmental stage at which this pattern occurs. We have now provided a new figure in the supplement in which we show the effects for each line/ combination of lines. Since we agree with the reviewer that the connection to the expression changes of reproduction-associated genes is tentative, we have now clearly separated results from discussion with the proposed mechanism. We also expanded the discussion to explain our rationale more clearly and we also discuss other hypotheses. Nevertheless, the observed difference between conspecific and heterospecific matings is very interesting-irrespectively of the underlying mechanism. It is highly original as this is the first time that a pattern that matches the predictions of mutation-order speciation and emerges from standing genetic variation.

5) There are some places in the manuscript where I think "progenies" should be "progeny" (l. 332, Y-axis of Fig 4b).

Thanks, we fixed this.


Reviewer #2:
This is an interesting study on the potential evolution of partial reproductive isolation associated with experimental evolution in a challenging environment. However, the present manuscript has several substantial flaws and considerable revision would be needed to reach publishability.

Positives:
* The idea of looking at reproductive isolation among experimentally evolved populations is very promising and I have wondered if/when the lab would turn to that topic.
* The evidence for (partial) pre-mating isolation, in terms of male courtship preference, is clear, and it does have some general relevance to the ecological speciation model.
* The integration of gene expression analyses with reproduction-oriented experiments provides complementary perspectives.
* The text is mostly clear and well-written, with some exceptions noted.

Negatives:
* The evidence for (partial) post-mating isolation is rather limited.
* To the extent that post-mating isolation exists, the authors fail to adequately consider the potential for genetic drift to underlie it.
* The narrow framing of the study leads to notably poor scholarship. For example, there is basically no acknowledgment of previous studies on laboratory evolution and reproductive isolation.
* There is a fair amount of over-interpretation and over-simplification in this manuscript.

Out of typical expediency, I focus below exclusively on the areas where improvement is needed.


Major points:

1. The authors assume that only natural selection can lead to reproductive isolation. They begin the Introduction by presenting a highly debatable claim as if it were a universally-acknowledged fact: "Most new species are formed by selection [1-3] as already suggested by Darwin [4]." Then, they proceed to frame their study strictly in terms of two specific models of selection-driven reproductive isolation: ecological speciation and mutation-order speciation. Another alternative the authors really must acknowledge is genetic drift. Drift makes the same prediction as mutation-order speciation (i.e. that reproductive isolation may occur between any populations, regardless of environment). The authors previously estimated that their experimental populations had an effective population size of around 300. The experiments reported here were conducted at various generations, between 100 and 200 generations after the lab populations were assembled. Hence, we are talking about
roughly 0.3-0.7Ne generations, which implies powerful drift (in addition to powerful selection). Schiffman & Ralph (2022) described conditions in which incompatibilities could arise neutrally in a scenario such as this. Quoting from their abstract: "system drift, is expected to proceed at a rate proportional to the amount of intrapopulation genetic variation divided by the effective population size (⁠Ne⁠). At biologically reasonable parameter values this could lead to substantial interpopulation incompatibility, and thus speciation, on a time scale of Ne generations.". In these experiments, there is a ton of variation going into the heat-exposed populations (from a highly diverse natural fly population), and yet lab Ne is small, creating a large ratio of variation to Ne, which entails far more favorable conditions for the system drift model than would exist in a constant-size natural population. Unfortunately, the authors do not seem to have any outbred control populations
subject to drift but not thermal selection, which might otherwise be useful in testing between the drift and mutation order hypotheses (although they would still experience lab adaptation).

The authors only belatedly mention drift in the latter part of their brief Discussion section. Their reasons for dismissing it revolve entirely around the expression patterns of reproductive genes in heat-evolted populations. However, they can not prove that the evolution of reproductive genes is related to the tentative signal of post-mating isolation they report (in other words, the expression patterns could be non-neutral but not contribute to post-mating isolation). Alternatively, even some of the evolution of reproductive genes could reflect drift, due to the reduced effectiveness of selection on sex-specific traits (Dapper & Wade 2020).

In the absence of any clearer evidence to differentiate these hypotheses, system drift must be placed on at least equal footing with mutation order speciation, including in the abstract.

We thank the reviewer for highlighting the potential of genetic drift for the emergence of reproductive isolation. We would like to emphasize, however, that the empirical evidence for this is sparse and older work has recently been challenged. In response to the points raised by the reviewer, we have now entirely re-structured our discussion and we discuss our model (selection for early fecundity plus sexual conflict) in comparison to the pure drift model proposed by the reviewer and a model of drift and sexual conflict.

Nevertheless, because empirical evidence for drift related speciation mechanisms is so controversial, we preferred to stick to the generally accepted view that most speciation is the outcome of selection.

Finally, we like to stress that the emergence of reproductive isolation among replicate populations from standing genetic variation is a real novelty, irrespective of whether this is driven by adaptation or it reflects a neutral process.

2. The current manuscript is completely bereft of relevant context from prior laboratory evolution studies. There is an entire branch of literature on the evolution of reproductive isolation in laboratory settings that is totally missing from this manuscript. As a starting point, I have copied several links at the bottom of my review, but there are almost certainly other relevant references out there as well. Such background must be summarized in the Introduction for the manuscript to reach a publishable state. Other areas of the literature are under-cited as well, including the roles of CHCs in Drosophila ecological adaptation and reproductive isolation.

Many thanks for the literature suggestions that we have largely incorporated into the revised manuscript.

3. The Results arguing for postmating isolation are limited and not especially convincing. The authors look at the numbers of F1 progeny from crosses within vs. between the 3 heat-selected replicate populations. They show only a coarse summary of these results (they should be presented in more detail in a supplemental table), which entail a modestly (8%) lower reproductive rate for the between-population crosses. The authors report a modestly significant P value (0.03) for this difference, although given the diallel design and the 5 replicates per cross, I'm not sure the assumptions of the Wilcoxon test are met. There is also ambiguity about what was actually done. This statement from the Results makes it sound like they looked at the offspring of F1s instead...
"F1 flies from crosses between replicates of the evolved 148 populations produced 8.3% fewer viable offspring"
...but the Methods text implies that they instead looked at F1 offspring of the crossed parental populations. Assuming the latter is correct, I am puzzled the authors did not look at the viability or reproductive success of F2 offspring, which are much more likely to manifest incompatibilities than F1s.

Many thanks for pointing this out! Of course, we did not study the offspring of the F1 flies, but the F1 directly. This has been fixed. We also provide a new figure in which we show the results of each of the crosses separately. Nevertheless, the data are noisy, therefore we did not test individual crosses.

4. Related to the above, there is a lot of simplistic and over-confident interpretation throughout the manuscript.

We agree that for better readability out text was simplistic. We have now revised the manuscript to make it clearer what we observe and what our interpretation of the observation is.

Examples:

Abstract: "Gene expression analysis identified the underlying molecular mechanisms"
Over-statement - no causal demonstration of mechanism is reported.

Correct-this is now changed

Abstract: "Premating ecological speciation is the byproduct of an altered lipid metabolism"
Falsely implies that speciation has fully occurred here.

Point taken, we modified the sentence

Intro: "We find support for ecological speciation"
As with the abstract, some nuance is needed here to avoid suggesting that complete speciation has occurred. "support for the ecological speciation model" would be more appropriate.

modified

Disc: "the pleiotropic effects of involved genes explain the premating reproductive isolation"
This is a promising hypothesis but it was not formally demonstrated here.

modified

Disc: "Remarkably, we did not only find evidence for ecological speciation, but also for mutation order speciation in the same experiment"
Same issues as above.

modified

And throughout the manuscript, the term "reproductive isolation" is used rather loosely. It might be interpreted as meaning "these fly populations are now reproductively isolated", when in fact the claim is just that there is a non-zero level of pre-mating and post-mating isolation between them. More careful language is called for.

We agree that the term “reproductive isolation” can be mis-read, but since even two Drosophila species, such as D. simulans and D. mauritiana are full reproductively isolated, we anticipate that the reader is aware that reproductive isolation is not complete-and the numbers in our results show this as well. Finally, the term reproductive isolation and incipient speciation are widely used in the community in the context of effects that are of similar strength as the ones in our study.

To make it clear that we do not talk about complete reproductive isolation, we added the term (incomplete) when we mention reproductive isolation for the first time. We hope that this avoids a misconception by the readers.


Specific things:

I agree with the authors that mutation-order speciation is conceptualized too narrowly and that standing variation and multiple loci is a more broadly relevant scenario. An alternative term that more generally invokes "contingency" in adaptation might be an improvement to the terminology of the field.

Figure 1 could be a starting point for a graphical abstract, but it doesn't really add much as a main paper figure.

We agree and have removed the figure from the manuscript

Figure 2A's axis labels and legend description are vaguely/ambiguously presented. Please clarify the males and females involved in each experiment. It's also not clear why there need to be "post-hoc groups". In Figure 2B, while I can guess what A and H might refer to, they aren't defined in the legend. The nature of the experiment and the conceptual meaning of the metric should also be spelled out in the legend.

We have improved the figure legend for more clarity.

Data Availability should be improved at this stage:
"Additional scripts and raw data are available on Github upon publication."
This is not a publishable statement. The authors need to clearly present what they intend to share publicly, so that reviewers can determine whether it is sufficient.

Sorry, we follow a widely used convention, but as the reviewer is interested to have a look-here is the link to the requested material:

<https://github.com/ShengKaiHsu/Dsim_reproductiveIsolation>

**Second round of review**

**Reviewer 1**

In this revised manuscript, the authors have been more cautious in their presentation and conclusions. They have also considered alternative hypotheses. Their conclusions are now better justified by the results. There are still a few points, mostly minor, that could be improved.

1) title: I think "arises" should be "arise"

2) line 52: "Most new species are formed by selection [1-3] as already suggested by Darwin [4]." - like the other reviewer, I find this statement to be rather bold and potentially controversial. It is probably not the best way to start the paper, especially because the authors' conclusions don't depend on this statement being true. Maybe qualify the statement slightly like "Adaptation is thought to play an important role in speciation, as suggested by Darwin"

3) line 58: "another" should be "other"

4) line 61: "and also" could simply be "and"

5) line 132: "We used gene expression data of the focal populations [21]" - it is not clear what data were used and what is meant by "focal populations". Perhaps the reader could figure this out by checking reference 21, but I think it would help to provide more detail in the methods section. Currently, the methods section is rather vague. Which evolved population replicates were used in the RNA-seq analysis? Are they the same that were used in the tests of reproductive isolation and analysis of CHCs? Or are the authors comparing results from different subsets of replicates? In line 396 they mention that they used only male samples. How is it that they have female expression data in Figure 2c? Maybe they are only referring to their analysis of reproduction-related genes in the methods? If so, they should make it clear that they are referring to "male reproduction-related genes" or "male-expressed reproduction-related genes" at several places in the manuscript (abstract, introduction, results, discussion, figure 4b). Their sexual conflict hypothesis would predict also an enrichment of female reproduction-related genes that vary in expression among replicates.

6) Do replicates of the base population also show greater heterogeneity in expression of male reproductive genes than background genes? If so, greater heterogeneity among evolved replicates would be expected by genetic drift.

7) line 191: "Remarkably, the patterns of postmating reproductive isolation follow the predictions of mutation-order speciation, the second mechanism of adaptive speciation." - I think the remarkable thing is that postmating isolation is detected at all between the replicates. Given that it is detected, one would expect it to be the second mechanism (of the two presented by the authors), as the first mechanism (ecological speciation) could not occur among replicates in the same environment. As the authors mention later, their observation also follows the predictions of genetic drift.

8) line 256: "and could not found for other" -> "and could not be found for other"

**Reviewer 2**

I appreciate that the authors have made some improvements to their manuscript. However,
the text remains deeply problematic in its framing...

Starting with the abstract, the text still focuses on the same two selective-driven speciation models. In my original review, I pointed out that the authors' experiment is within a parameter space (high diversity, low Ne, roughly ~0.5Ne generations) in which modeling has shown that genetic drift should have a strong potential to generate reproductive isolation. I am not at all satisfied with the authors' response on this subject. They label the idea of drift leading to reproductive isolation as being somehow too controversial to merit any major focus. Even if that was accurate, do controversial topics need not be addressed in science? And besides, neutral evolution is traditionally considered a null hypothesis in evolutionary genetics. Hence, a predominant focus on adaptive processes is only appropriate if the authors can reject neutral explanations. They suggest that there is limited empirical evidence for drift leading to reproductive isolation. Clearly, the roles of drift and selection are inherently difficult to disentangle in cases obvious ecological differences are not present. But clearly there are countless pairs of related taxa with no obvious ecological differences. And more generally, I would suggest that the relative role of drift vs. selection in speciation is considered very much an open question (e.g. Cutter 2011; Sweigart & Willis 2012; Gavrilets 2014) - in stark contrast to this manuscript's Introduction and the framing of its own results. Furthermore, empirical evidence for mutation-order speciation is also very limited, which undermines any rationale for ascribing it an a-priori preference over drift. And regardless of what is or isn't happening in nature, it is clear that drift is predicted to be a potent force in the present lab experiment (see above, and my previous review). In the present context, drift may be less of a concern for the evolution of premating isolation in light of previous experimental results (e.g. Rundle 2003; Matute 2013). However, the systems drift model is highly relevant to postmating isolation, which is where the authors' evidence is weaker anyway...

In my previous review, I pointed out that the authors' claim for postmating isolation is based on a test that may not be valid ("given the diallel design and the 5 replicates per cross, I'm not sure the assumptions of the Wilcoxon test are met"). The authors did not respond to this concern. They also did not address my request for replicate-level fecundity data to be given in a supplemental table. Hence, I am not satisfied that the authors have demonstrated clear evidence for postmating isolation.

Thus, there are two separate major problems with the authors' conclusions regarding postmating isolation and mutation-order speciation. First, the evidence for the existence post-mating isolation in their populations is not very convincing. Second, any post-mating isolation that does exist could easily be a product of drift rather than selection. For the manuscript to reach a publishable stage, the authors would need to revise their abstract and main text accordingly - softening their claims of post-mating isolation ("potential evidence" seems more appropriate), and equally mentioning drift and mutation-order speciation as potential explanations. These changes would still allow for an interesting manuscript that could be framed more heavily around its evidence for ecological speciation. I truly think a more even-handed manuscript of this sort would receive more respect from the scientific community than the present version.

**Authors’ response to reviewers**

Reviewer #1: In this revised manuscript, the authors have been more cautious in their presentation and conclusions. They have also considered alternative hypotheses. Their conclusions are now better justified by the results. There are still a few points, mostly minor, that could be improved.

We appreciate the positive feedback.

1) title: I think "arises" should be "arise"

Thanks for pointing out the grammatical error. This is fixed in the revised manuscript.

2) line 52: "Most new species are formed by selection [1-3] as already suggested by Darwin [4]." - like the other reviewer, I find this statement to be rather bold and potentially controversial. It is probably not the best way to start the paper, especially because the authors' conclusions don't depend on this statement being true. Maybe qualify the statement slightly like "Adaptation is thought to play an important role in speciation, as suggested by Darwin"

We modified the sentence according to the reviewer’s suggestion. In addition, we re-framed the introduction with more equal-handed views to the both effects of selection and drift during speciation.

3) line 58: "another" should be "other"

Thanks for pointing out the grammatical error. This is fixed in the revised manuscript.

4) line 61: "and also" could simply be "and"

Thanks for pointing out the grammatical error. This is fixed in the revised manuscript.

5) line 132: "We used gene expression data of the focal populations [21]" - it is not clear what data were used and what is meant by "focal populations". Perhaps the reader could figure this out by checking reference 21, but I think it would help to provide more detail in the methods section. Currently, the methods section is rather vague. Which evolved population replicates were used in the RNA-seq analysis? Are they the same that were used in the tests of reproductive isolation and analysis of CHCs? Or are the authors comparing results from different subsets of replicates? In line 396 they mention that they used only  male samples. How is it that they have female expression data in Figure 2c? Maybe they are only referring to their analysis of reproduction-related genes in the methods? If so, they should make it clear that they are referring to "male reproduction-related genes" or "male-expressed reproduction-related genes" at several places in the manuscript (abstract, introduction, results, discussion, figure 4b). Their sexual conflict hypothesis would predict also an enrichment of female reproduction-related genes that vary in expression among replicates.

Thanks for pointing out the uncertainty. We provide now more details about the origin of the RNA-Seq data (Ln. 221-224 & Ln. 255). These gene expression data as well as the CHC assays were collected for all independently evolved replicates. The mating assays were done only for a subset of the evolved replicates as mentioned in Ln. 578. To study the gene expression changes of CHC-related genes during evolution (Figure 2c), we used the expression difference between the evolved and ancestral populations from a supplementary table in Hsu et al. (2020), which is already mentioned as the source of the RNA-Seq data. Because this data set contained three replicates for each of the evolved replicates, we could use it to investigate the difference among independently evolved replicates with the hypothesis that causal genes underlying the putative postmating isolation among replicates would diverge more in gene expression. This analysis is limited to male samples as explained in Ln. 654-655.

6) Do replicates of the base population also show greater heterogeneity in expression of male reproductive genes than background genes? If so, greater heterogeneity among evolved replicates would be expected by genetic drift.

The five replicates of the base population are expected to be genetically identical so little variance in expression is expected. To answer the reviewer’s question, we made a new analysis comparing the expression variance (measured as coefficient of variation) between the reproductive genes and the background gene sets and observed no significant difference (See attached figure; Wilcoxon’s test, p = 0.365).


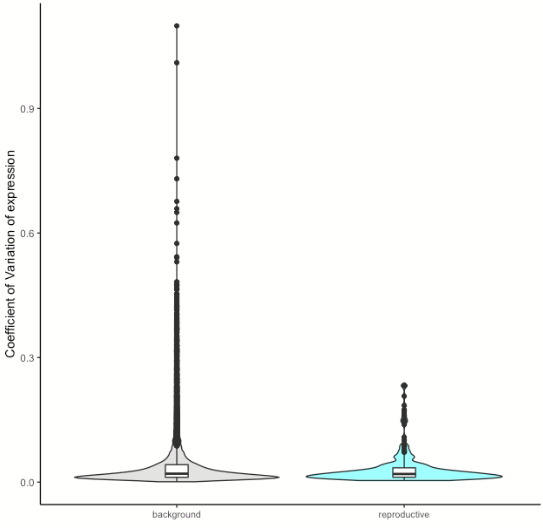


7) line 191: "Remarkably, the patterns of postmating reproductive isolation follow the predictions of mutation-order speciation, the second mechanism of adaptive speciation." - I think the remarkable thing is that postmating isolation is detected at all between the replicates. Given that it is detected, one would expect it to be the second mechanism (of the two presented by the authors), as the first mechanism (ecological speciation) could not occur among replicates in the same environment. As the authors mention later, their observation also follows the predictions of genetic drift.

We removed this sentence in the revised manuscript.

8) line 256: "and could not found for other" -> "and could not be found for other"

Thanks for pointing out the grammar error. This is fixed in the revised manuscript.

Reviewer #2: I appreciate that the authors have made some improvements to their manuscript. However, the text remains deeply problematic in its framing...

Starting with the abstract, the text still focuses on the same two selective-driven speciation models.  In my original review, I pointed out that the authors' experiment is within a parameter space (high diversity, low Ne, roughly ~0.5Ne generations) in which modeling has shown that genetic drift should have a strong potential to generate reproductive isolation.  I am not at all satisfied with the authors' response on this subject.  They label the idea of drift leading to reproductive isolation as being somehow too controversial to merit any major focus.  Even if that was accurate, do controversial topics need not be addressed in science?  And besides, neutral evolution is traditionally considered a null hypothesis in evolutionary genetics.  Hence, a predominant focus on adaptive processes is only appropriate if the authors can reject neutral explanations.  They suggest that there is limited empirical evidence for drift leading to reproductive isolation.  Clearly, the roles of drift and selection are inherently difficult to disentangle in cases obvious ecological differences are not present.  But clearly there are countless pairs of related taxa with no obvious ecological differences.  And more generally, I would suggest that the relative role of drift vs. selection in speciation is considered very much an open question (e.g. Cutter 2011; Sweigart & Willis 2012; Gavrilets 2014) - in stark contrast to this manuscript's Introduction and the framing of its own results.  Furthermore, empirical evidence for mutation-order speciation is also very limited, which undermines any rationale for ascribing it an a-priori preference over drift.  And regardless of what is or isn't happening in nature, it is clear that drift is predicted to be a potent force in the present lab experiment (see above, and my previous review).  In the present context, drift may be less of a concern for the evolution of premating isolation in light of previous experimental results (e.g. Rundle 2003; Matute 2013).  However, the systems drift model is highly relevant to postmating isolation, which is where the authors' evidence is weaker anyway...

We acknowledge the reviewer’s concern and rewrote the manuscript with a more even-handed framework to the effects of both drift and selection. We added a new introduction paragraph to review the literature on the development of system drift model and discussed both selection and drift hypotheses equally. Correspondingly, the abstract was also rewritten.

In my previous review, I pointed out that the authors' claim for postmating isolation is based on a test that may not be valid ("given the diallel design and the 5 replicates per cross, I'm not sure the assumptions of the Wilcoxon test are met").  The authors did not respond to this concern.  They also did not address my request for replicate-level fecundity data to be given in a supplemental table.  Hence, I am not satisfied that the authors have demonstrated clear evidence for postmating isolation.

It is not clear to us what assumption the reviewer was concerning. Is it the independence of samples? We did check for the dependence among the 5 replicates for each cross using a linear mixed effect model and found no significant dependence. Hence, we treated them as independent replicates in the Wilcoxon test. We clarified this in the revised manuscript (Ln. 641-644). On the replicate-level data, we provide the results in a supplementary figure (Figure S3).

Thus, there are two separate major problems with the authors' conclusions regarding postmating isolation and mutation-order speciation.  First, the evidence for the existence post-mating isolation in their populations is not very convincing.  Second, any post-mating isolation that does exist could easily be a product of drift rather than selection.  For the manuscript to reach a publishable stage, the authors would need to revise their abstract and main text accordingly - softening their claims of post-mating isolation ("potential evidence" seems more appropriate), and equally mentioning drift and mutation-order speciation as potential explanations.  These changes would still allow for an interesting manuscript that could be framed more heavily around its evidence for ecological speciation.  I truly think a more even-handed manuscript of this sort would receive more respect from the scientific community than the present version.

We really appreciate the reviewer’s suggestion on how to improve the manuscript. In the revised version, we avoid definitive wording on our claim of postmating isolation and equally mentioned mutation-order speciation and system drift as potential explanations.

**Third round of review**

**Reviewer 2**

I am mostly satisfied with the latest set of revisions in response to my previous round of comments. A few final points requiring attention:

The abstract took my suggestion to refer to *potential* evidence of postmating isolation. Another place such rewording is needed is in the legend of Figure 3. Also, the TITLE of the paper is still "Pre- and postmating reproductive isolation arise during adaptation to a novel hot environment", which still implies unambiguous evidence for both types of isolation individually. One example of an alternative title that avoids this implication is: "Reproductive isolation arises during laboratory adaptation to a novel hot environment".

The concern I mentioned in my first two reviews about the Wilcoxon test in the postmating isolation analysis was indeed with regard to violations of the assumption of independence. Here there is a nested experimental structure in which multiple replicates are studied from each distinct cross. The authors' text currently states:
"We used a linear mixed effect model to determine whether the number of viable progenies was correlated among crosses involving independent flies for the same crossing scheme. Since we did not detect a correlation, we treated all replicated crosses as independent observations."
This is a strange statistical practice. Given that in the mixed model approach, the cross effect was not statistically significant (unclear how close it might have been), the authors then moved to a test that assumes no cross effect at all (Wilcoxon), and there they get a just-significant result. The more conventional/sound approach would be to to simply use the mixed model approach to test for the environmental difference, acknowledging that even if a cross effect is not significant on its own, it may well exist. Given that the authors ran the mixed model, it seems likely that they have already observed this result, and that it was non-significant. And so, I remain concerned that evidence for postmating isolation is exaggerated as currently reported.

**Authors’ response to reviewers**

Reviewer #2: I am mostly satisfied with the latest set of revisions in response to my previous round of comments. A few final points requiring attention: The abstract took my suggestion to refer to *potential* evidence of postmating isolation. Another place such rewording is needed is in the legend of Figure 3. Also, the TITLE of the paper is still "Pre- and postmating reproductive isolation arise during adaptation to a novel hot environment", which still implies unambiguous evidence for both types of isolation individually. One example of an alternative title that avoids this implication is: "Reproductive isolation arises during laboratory adaptation to a novel hot environment".

WE CHANGED THE TITLE ACCORDING TO THE REVIEWER’S SUGGESTION.

The concern I mentioned in my first two reviews about the Wilcoxon test in the postmating isolation analysis was indeed with regard to violations of the assumption of independence. Here there is a nested experimental structure in which multiple replicates are studied from each distinct cross. The authors' text currently states: "We used a linear mixed effect model to determine whether the number of viable progenies was correlated among crosses involving independent flies for the same crossing scheme. Since we did not detect a correlation, we treated all replicated crosses as independent observations." This is a strange statistical practice. Given that in the mixed model approach, the cross effect was not statistically significant (unclear how close it might have been), the authors then moved to a test that assumes no cross effect at all (Wilcoxon), and there they get a just-significant result. The more conventional/sound approach would be to to simply use the mixed model approach to test for the environmental difference, acknowledging that even if a cross effect is not significant on its own, it may well exist. Given that the authors ran the mixed model, it seems likely that they have already observed this result, and that it was non-significant. And so, I remain concerned that evidence for postmating isolation is exaggerated as currently reported.

WE ACKNOWLEDGE THE REVIEWER’S CAUTION ON THIS ISSUE. WE INCLUDED THE P-VALUE OF THE EFFECT ON INTER- AND INTRA-POPULATION CROSSES IN THE MIXED MODEL IN THE REVISED MANUSCRIPT AND CLARIFY THE REASONABLE TO MOVE FROM A TWO-TAIL ANOVA TEST TO ONE-TAIL WILCOXON’S TEST.
